# DDHD2 provides a flux of saturated fatty acids for neuronal energy and function

**DOI:** 10.1038/s42255-025-01367-x

**Published:** 2025-09-30

**Authors:** Saber H. Saber, Nyakuoy Yak, Xuan Ling Hilary Yong, Yih Tyng Bong, Hannah Leeson, Chuan-Yang Dai, Tobias Binder, Siyuan Lu, Reshinthine Purushothaman, An-Sofie Lenaerts, Leonardo Almeida-Souza, Lidiia Koludarova, Safak Er, Irena Hlushchuk, Arnaud Gaudin, Sachin Singh, Tuula A. Nyman, Jeffrey R. Harmer, Steven Zuryn, Ernst Wolvetang, Gert Hoy Talbo, Mikko Airavaara, Brendan J. Battersby, Ashley J. van Waardenberg, Victor Anggono, Giuseppe Balistreri, Merja Joensuu

**Affiliations:** 1 Australian Institute for Bioengineering and Nanotechnology, The University of Queensland, Brisbane, Queensland Australia; 2 Queensland Brain Institute, Faculty of Health, Medicine and Behavioural Sciences, The University of Queensland, Brisbane, Queensland Australia; 3 Zoology and Entomology Department, Faculty of Science, Assiut University, Assiut, Egypt; 4 Clem Jones Centre for Ageing Dementia Research, The University of Queensland, Brisbane, Queensland Australia; 5 Institute of Biotechnology, Helsinki Institute of Life Science, University of Helsinki, Helsinki, Finland; 6 Helsinki Institute of Life Science (HiLIFE), University of Helsinki, Helsinki, Finland; 7 Faculty of Biological and Environmental Sciences, University of Helsinki, Helsinki, Finland; 8 Drug Research Program, Division of Pharmacology and Pharmacotherapy, Faculty of Pharmacy, University of Helsinki, Helsinki, Finland; 9 Department of Immunology, Oslo University Hospital and University of Oslo, Oslo, Norway; 10 Centre for Advanced Imaging, The University of Queensland, Brisbane, Queensland Australia; 11 NHMRC Centre for Research Excellence in Mechanisms in NeuroDegeneration – Alzheimer’s Disease (MIND-AD CRE), Brisbane, Queensland Australia; 12 i-Synapse PTY LTD, Cairns, Queensland Australia; 13 Department of Virology, Faculty of Medicine, University of Helsinki, Helsinki, Finland

**Keywords:** Diseases of the nervous system, Cellular neuroscience, Learning and memory, Metabolism, Neurophysiology

## Abstract

Although fatty acids support mitochondrial ATP production in most tissues, neurons are believed to rely exclusively on glucose for energy. Here we show that genetic ablation of the triglyceride and phospholipid lipase *Ddhd2* impairs mitochondrial respiration and ATP synthesis in cultured neurons, despite increased glycolysis. This defect arises from reduced levels of long-chain saturated free fatty acids, particularly myristic, palmitic and stearic acids, normally released in an activity-dependent manner by Ddhd2. Inhibition of mitochondrial fatty acid import in wild-type neurons similarly reduced mitochondrial respiration and ATP production. Saturated fatty acyl-coenzyme A treatment restored mitochondrial energy production in *Ddhd2* knockout neurons. When provided in combination, these activated fatty acyl-CoA supplements also rescued defects in membrane trafficking, synaptic function and protein homeostasis. These findings uncover that neurons perform β-oxidation of endogenous long-chain free fatty acids to meet ATP demands and reveal a potential therapeutic strategy for hereditary spastic paraplegia 54 caused by *DDHD2* mutations.

## Abstract (web summary)

Saber et al. show that the lipase DDHD2 provides endogenous saturated fatty acids to support fatty acid oxidation and energy production, proteostasis and membrane trafficking balance.

## Main

DDHD2 is a mammalian intracellular phospholipase A1 that cleaves acyl ester bonds from phospholipids and triglycerides^1–3^, generating saturated free fatty acids (sFFAs) and 2-acyl-lysophospholipids^3–5^. Biallelic mutations in *DDHD2* can disrupt its membrane-binding domain and abolish phospholipase and triglyceride hydrolase activities, causing hereditary spastic paraplegia 54 (HSP54), a childhood-onset autosomal recessive disorder marked by progressive neuromuscular and cognitive impairments^6–15^. The mechanisms linking *DDHD2* mutations to HSP54 remain unclear, and no cure or effective treatment currently exists. Consistent with its role in lipid metabolism, loss of DDHD2 function leads to lipid accumulation in human HSP54 brains^6^ and lipid droplet build-up in *Ddhd2* knockout (*Ddhd2*^−/−^)^1^ mouse neurons^1,2^. The lipid droplet increase in neurons is paradoxical, as lipid droplets are primarily considered to be a fuel storage of fats, and neurons are thought to rely mainly on glucose or astrocyte-derived metabolites for energy^16^.

We recently found that Ddhd2 releases specific sFFAs, particularly long-chain myristic (C14:0), palmitic (C16:0) and stearic (C18:0) acids, in an activity-dependent manner, both in neuronal cultures following stimulation in vitro, and in vivo in the brains of healthy *Ddhd2*^+/+^ mice following energy-demanding learning and memory behavioural tests^3^. In contrast, *Ddhd2*^−/−^ mice, which exhibit progressive cognitive and neuromuscular decline resembling HSP54, showed reduced basal sFFA levels across brain regions, including the hippocampus, compared to control mice before the onset of symptoms^3^. These differences were further exacerbated following neuronal activity^3^.

Here, we show that the flux of intracellular sFFAs myristic, palmitic and stearic acids released by Ddhd2 fuel mitochondrial fatty acid β-oxidation to support energy production alongside glycolysis both at basal conditions and following neuronal stimulation. Loss of this pathway in *Ddhd2*^−/−^ neurons reduced acetyl coenzyme A (acetyl-CoA) levels, a key citric acid (Krebs) cycle metabolite and, similarly to acute pharmacological inhibition of mitochondrial fatty acid import in wild-type neurons, impaired mitochondrial respiration and ATP production. Combined with evidence that cortical neurons express the full β-oxidation and carnitine cycle machinery, our findings reveal that approximately 20% of neuronal basal energy is derived from the Ddhd2-dependent β-oxidation pathway and that this energy pathway is particularly important under energy-demanding conditions. Notably, 48-h treatment with coenzyme A (CoA)-conjugated myristic, palmitic and stearic acids (M-CoA, P-CoA and S-CoA, respectively) restored mitochondrial respiration and ATP production in *Ddhd2*^−/−^ neurons and in neurons expressing an HSP54-associated DDHD2 mutant, without inducing oxidative stress. When combined, but less efficiently when supplemented alone, these activated fatty acids efficiently rescued defects in membrane trafficking, mitochondrial structure and distribution, synaptic function and proteostasis, highlighting a potential therapeutic strategy for HSP54, and suggesting that the Ddhd2-mediated release of myristic, palmitic and stearic acids serves additional functions in neurons beyond bioenergetics. Together, our findings demonstrate that mitochondrial β-oxidation is a critical energy source for neurons, particularly during high activity.

## Results

### Ddhd2 loss reduces cellular ATP levels despite enhanced glycolysis

**Fig. 1:**
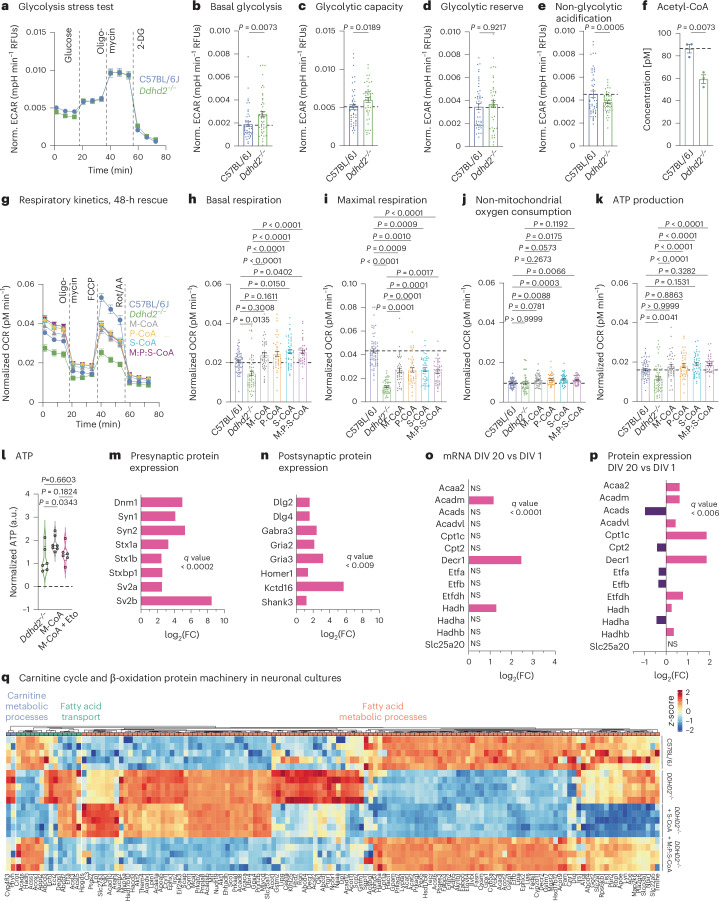
β-oxidation pathway for mitochondrial energy production in neurons. **a**, Seahorse XF measurement of ECAR kinetics in embryonic day (E) 16 C57BL/6J and *Ddhd2*^−/−^ neuronal cultures at DIV 21–22. Injection of glucose, oligomycin and 2-DG is indicated. Quantification of ECAR. **b**–**e**, Basal glycolysis (**b**), glycolytic capacity (**c**), glycolytic reserve (**d**) and non-glycolytic (**e**) acidification in C57BL/6J and *Ddhd2*^−/−^ neurons. **f**, Fluorometric acetyl-CoA quantification in adult C57BL/6J and *Ddhd2*^−/−^ brain lysates. **g**, Seahorse XF OCR. **h**–**k**, Quantification of basal respiration (**h**), maximal respiration (**i**), non-mitochondrial oxygen consumption (**j**) and ATP production (**k**) in DIV 21–22 hippocampal neuron cultures of C57BL/6J, and *Ddhd2*^−/−^ ± 1 µM M-CoA, P-CoA, S-CoA, or 1 µM M:P:S-CoA for 48 h. Injection of oligomycin, FCCP and Rot/AA are indicated. **l**, Luminescence detection of intracellular ATP (a.u., arbitrary units) in cultured hippocampal E16 *Ddhd2*^−/−^ neurons ±1 µM M-CoA for 48 h and ± etomoxir analysed at DIV 21–22 normalized to the average of *Ddhd2*^−/−^ in each experiment. **m**,**n**, LFQ LC–MS/MS protein expression analysis of presynaptic (**m**) and postsynaptic (**n**) proteins from E16 cultured NMRI mouse cortical neuron–glia cultures at DIV 20 versus DIV 1. **o**, DESeq2 comparison of gene expression changes for mRNAs encoding enzymes of the mitochondrial carnitine cycle and β-oxidation at DIV 20 versus DIV 1 from cultured E16 NMRI mouse cortical neurons. **p**, LFQ LC–MS/MS expression analysis for mitochondrial carnitine cycle and β-oxidation proteins at DIV 20 versus DIV 1 from cultured E16 NMRI mouse cortical neurons. **q**, LFQ LC–MS/MS protein abundance analysis of fatty acid metabolic processes (GO:0006631), fatty acid transport (GO:0015908) and carnitine metabolic processes (GO:0009437) in cortical neuron cultures of E16 C57BL/6J, *Ddhd2*^−/−^ ± 1 µM S-CoA or 1 µM M:P:S-CoA for 48 h and analysed at DIV 21–22. The expression heat map shows column-wise *z*-score-normalized protein abundances in each condition with each column representing a unique protein and each row a biological replicate. Samples and proteins were clustered using Euclidean distance and complete linkage. The colour gradients in heat maps reflect *z*-scores from low (blue) to high (red) abundance. Dot plots and kinetic curves are presented as the mean ± s.e.m.; violin plot is median ± quartiles. Dots present technical replicates. *N* = 3 (**a**–**i**) and *N* = 5 (**m**–**q**) biologically independent experiments in each condition. The exact *P* values stated in the graphs were determined from biological replicates using a two-tailed unpaired *t*-test (**b**, **d**, **e**, **f** and **g**), two-tailed Mann–Whitney test (**c**), ordinary one-way analysis of variance (ANOVA) Kruskal–Wallis multiple-comparison test (**h**, **i** and **k**), ordinary one-way ANOVA Sidak’s multiple-comparisons test (**j**) and ordinary one-way ANOVA Tukey’s multiple-comparisons test (**l**). FC, fold change; NS, not significant. RFU, relative fluorescence unit.

**Extended Data Fig. 1:**
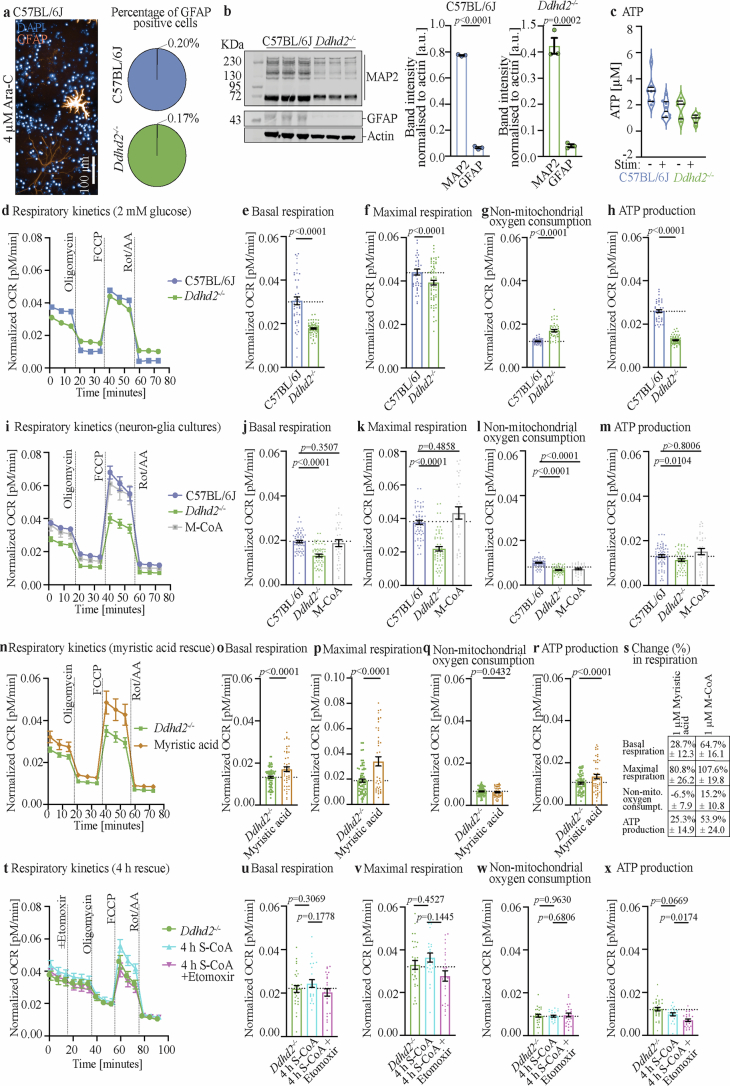
Mitochondrial respiration and glycolysis in neuronal cultures. **a**, Representative immunofluorescence image of endogenous GFAP staining in E16 C57BL/6J hippocampal neuron cultures treated with 4 µM Ara-C from DIV3 onwards until DIV21, after which the cultures were fixed for immunofluorescence staining. Quantification shows percentage (%) of GFAP-positive cells normalised to DAPI count in 4 µM Ara-C-treated (DIV 3-21) C57BL/6J and *Ddhd2*^*−/−*^ cultures. **b**, Western blot and quantification of E16 C57BL/6J and *Ddhd2*^*−/−*^ cultures treated with 4 µM Ara-C-treated at DIV3 to DIV21, and immunostained for MAP2, GFAP, and actin. MAP2 and GFAP band intensity are shown normalised to actin in C57BL/6J and *Ddhd2*^*−/−*^. **c**, Luminescence detection of intracellular ATP levels [µM] in cultured hippocampal E16 C57BL/6J and *Ddhd2*^*−/−*^ neuronal cultures at DIV21–22, measured at rest (–) and after 5 min high K⁺ stimulation (+). Seahorse XF OCR measurements in E16 **d-h**, hippocampal neuron cultures of C57BL/6J and *Ddhhd2*^*−/−*^ ( + 4 µM Ara-C) incubated in culture media containing 2 mM glucose for 1 h prior OCR measurements, **i-m**, E16 neuron-glia cultures (no Ara-C) of C57BL/6J, and *Ddhd2*^*−/−*^ ± 1 µM M-CoA for 48 h, **n-s**, E16 hippocampal neuron cultures ( + 4 µM Ara-C) of C57BL/6J and *Ddhd2*^*−/−*^ ± 1 µM myristic acid for 48 h, and the comparison of the percentage (%) change in mitochondrial respiration in *Ddhd2*^*−/−*^ following a rescue with 1 µm myristic acid or 1µM M-CoA for 48 h (**s**, see Fig. 1g-k, for comparison with M-CoA rescue), and **t-x**, E16 hippocampal neuron cultures ( + 4 µM Ara-C) of C57BL/6J and *Ddhd2*^*−/−*^ ± 1 µM S-CoA for 4 h ± etomoxir, both in the presence of cycloheximide. All analysed at DIV 21-22. Respiration kinetics, basal respiration, maximal respiration, non-mitochondrial oxygen consumption, and ATP production are shown for each condition. Injection of oligomycin, FCCP, Rot/AA, and etomoxir are indicated. Data are presented as mean values ± SEM, dots present technical replicates. N = 3 biologically independent experiments in each condition (**b, d-x**,) and N = 2 (**c**,). The exact *p*-values stated in the graphs were determined from biological replicates using two-tailed unpaired *t* test (**b, e, h, o-r**,), two-tailed Mann-Whitney test (**f, g**,), ordinary one-way ANOVA Dunnett’s multiple comparisons test (**j**,), ordinary one-way ANOVA Kruskal-Wallis multiple comparisons test (**k-m**,), ordinary one-way ANOVA Šídák’s multiple comparisons test (**u, v, w, x**,). Source data

To investigate the role of Ddhd2 on neuronal energy production, intracellular ATP levels were measured in cultured hippocampal neurons from C57BL/6J (control) and *Ddhd2*^−/−^ mice at 21–22 days in vitro (DIV). To suppress glial proliferation, neuronal cultures were supplemented with 4 µM cytosine β-d-arabinofuranoside (Ara-C, from here onwards referred to as neuronal culture; Extended Data Fig. 1a,b). Chemiluminescence ATP detection assay showed that *Ddhd2*^−/−^ neurons had lower ATP levels than controls at rest, with further reduction following a 5-min high K^+^ stimulation (Extended Data Fig. 1c). To assess whether reduced ATP production stemmed from impaired glycolysis, we measured glycolytic function in C57BL/6J and *Ddhd2*^−/−^ hippocampal neurons using the Seahorse glycolysis stress test. Based on extracellular acidification rates (ECARs; Fig. 1a), *Ddhd2*^−/−^ neurons displayed significantly increased basal glycolysis (Fig. 1b) and glycolytic capacity (Fig. 1c), while glycolytic reserve remained unchanged (Fig. 1d) and non-glycolytic acidification reduced significantly (Fig. 1e). These results indicate that ATP reduction in *Ddhd2*^−/−^ neurons is not due to impaired glycolysis. Combined with our previous findings that Ddhd2 releases sFFAs in mouse neurons and brain tissues especially following neuronal activity^3^, this result suggests that loss of the sFFA fluxes in *Ddhd2*^−/−^ mouse neurons may underlie the reduced ATP production through impaired mitochondrial respiration.

### External fatty acyl-CoA restores mitochondrial respiration in *Ddhd2*^−/−^ neurons and is blocked by Cpt1 inhibition

To explore the possibility that loss of Ddhd2 causes impairment of mitochondrial respiration, acetyl-CoA levels were measured in C57BL/6J and *Ddhd2*^−/−^ brain tissues using a fluorometric assay. Acetyl-CoA, mainly produced in mitochondria from carbohydrates, amino acids and fatty acids, is central to cellular energy metabolism. *Ddhd2* depletion disrupts sFFA turnover and is therefore likely to reduce acetyl-CoA levels in *Ddhd2*^−/−^ brain tissue. As expected, *Ddhd2*^−/−^ brains showed a significant 31.5% reduction in acetyl-CoA levels compared to controls (Fig. 1f), suggesting impaired mitochondrial respiration.

To directly assess mitochondrial respiration, we measured the oxygen consumption rate (OCR) in cultured neurons using the Seahorse Cell Mito Stress Test. *Ddhd2*^−/−^ neurons exhibited reduced OCR (Fig. 1g), including significantly lower basal respiration (Fig. 1h) and maximal respiration (Fig. 1i) compared to controls under 10 mM glucose conditions. While the non-mitochondrial oxygen consumption was not altered (Fig. 1j), ATP levels decreased significantly (~20% decrease; Fig. 1k), confirming defective mitochondrial respiration in *Ddhd2*^−/−^ neurons. Similar significant reductions were observed in neurons incubated in cultured media containing a more physiological glucose concentration, 2 mM (Extended Data Fig. 1d–h), and in neuron–glia co-cultures (without Ara-C-treatment; Extended Data Fig. i–m), indicating that the observed ATP deficit and decreased OCR levels in *Ddhd2*^−/−^ neurons are not a metabolic adaptation to a high glucose concentration or caused by the absence of glial cells. C57BL/6J control neurons cultured in 2 mM glucose exhibited higher mitochondrial basal respiration and ATP production than those in 10 mM glucose, suggesting that abundant glucose downregulates mitochondrial activity. A similar trend was observed in *Ddhd2*^−/−^ neurons cultured in 2 mM glucose compared to 10 mM glucose (Extended Data Fig. 1d–h). Notably, *Ddhd2*^−/−^ neurons cultured in 10 mM glucose showed lower maximal respiration levels compared to neurons that were cultured in 2 mM glucose (Fig. 1i and Extended Data Fig. 1f), which could indicate differences in how neurons adapt to reduced glucose availability by increasing reliance on other energy sources and optimizing mitochondrial function. Furthermore, *Ddhd2*^−/−^ neurons showed significantly increased levels of non-mitochondrial oxygen consumption in 2 mM glucose conditions compared to control neurons, which may indicate metabolic stress and cellular attempts to maintain redox homeostasis. Together, these results suggest an inverse regulation between neuronal glycolysis and mitochondrial respiration.

We recently showed that Ddhd2 loss reduces sFFA levels, particularly myristic, palmitic and stearic acids, in *Ddhd2*^−/−^ mouse brains and cultured neurons^3^. These deficits were further exacerbated by in vivo learning and memory tasks, as well as by neuronal activity induction in vitro^3^. Therefore, we hypothesized that exogenous sFFA supplementation could compensate for Ddhd2 loss. Fatty acid activation by CoA, where FFAs are esterified to form fatty acyl-CoAs, is an essential prerequisite for their utilization in β-oxidation^17,18^, protein lipidation^19,20^ (N-myristoylation and S-palmitoylation) or complex lipid synthesis^21^. Supplementation with 1 µM M-CoA, P-CoA and S-CoA acids, alone or combined, for 48 h restored mitochondrial respiration and ATP production significantly in *Ddhd2*^−/−^ neurons (Fig. 1g–k). Similar restoration of mitochondrial respiration was also observed in *Ddhd2*^−/−^ neuron–glia cultures following 1 µM M-CoA treatment for 48 h (Extended Data Fig. 1n–r). By comparison, while 1 µM myristic acid improved mitochondrial ATP production by 28.7% (Extended Data Fig. 1n–r), the activated M-CoA increased ATP production by 64.7% (Extended Data Fig. 1s). To investigate if the restoration of neuronal ATP levels was due to restoration of mitochondrial β-oxidation, the impact of etomoxir^22^, a carnitine palmitoyltransferase 1 (Cpt1) inhibitor that blocks mitochondrial import of long-chain fatty acids, was investigated using luminescent ATP detection assay. Acute etomoxir treatment blocked the restoration of ATP levels in *Ddhd2*^−/−^ neurons supplemented with 1 µM M-CoA for 48 h (Fig. 1l).

To examine the time dependence of fatty acyl-CoA rescue of ATP levels, we used stearic acid, which, unlike palmitic and myristic acid, is not involved in protein lipidation. Experiments were conducted in the presence of cycloheximide to block de novo protein synthesis^23^. Acute rescue with 1 µM S-CoA for 4 h in *Ddhd2*^−/−^ neurons did not improve mitochondrial respiration and ATP levels (Extended Data Fig. 1t–x), indicating that longer supplementation is required for efficient bioenergetic rescue. However, in *Ddhd2*^−/−^ neurons treated for 4 h with 1 µM S-CoA, etomoxir markedly decreased OCR and significantly decreased ATP production (Extended Data Fig. 1t–x). Together, these results indicate that the reduced ATP and acetyl-CoA levels in *Ddhd2*^−/−^ neurons result from impaired Ddhd2-dependent sFFA responses, and that these energy deficits can be efficiently rescued by extracellular CoA-conjugated myristic, palmitic and stearic acids, suggesting that these long-chain fatty acids serve as fuel for neuronal β-oxidation.

### Disrupted mitochondrial fatty acid import and β-oxidation machinery in *Ddhd2*^−^^/^^−^ neurons is restored by M:P:S-CoA

**Extended Data Fig. 2:**
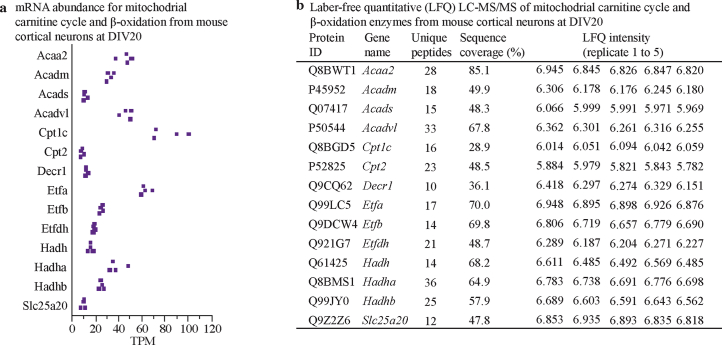
Mitochondrial carnitine cycle and β-oxidation enzymes expression in cultured cortical neurons. **a**, Total RNAseq expression of mRNAs encoding enzymes of the mitochondrial carnitine cycle and β-oxidation at DIV20 from cultured mouse cortical neurons. TPM, transcript per million. **b**, Label-free quantitative (LFQ) liquid chromatography-mass spectrometry (LC-MS/MS) expression analysis of the enzymes of the mitochondrial carnitine cycle and β-oxidation at DIV20 from cultured mouse cortical neurons. N = 5 biologically independent experiments in each condition.

To assess the capacity of neurons for mitochondrial fatty acid oxidation, we measured β-oxidation and carnitine cycle mRNA and protein levels in cultured mouse cortical neurons using quantitative PCR (qPCR) and label-free quantitative (LFQ) liquid chromatography–tandem mass spectrometry (LC–MS/MS), respectively. As neurons matured and formed synaptic connections (Fig. 1m,n), all key factors required for fatty acid mitochondrial import and oxidation were robustly expressed at both mRNA (Fig. 1o and Supplementary Table 1) and protein levels (Fig. 1p, Extended Data Fig. 2 and Supplementary Table 2). Notably, Cpt1c, which facilitates fatty acyl-CoA import into mitochondria and is a rate-limiting step in β-oxidation at times of high energy requirements^24–26^, was among the most upregulated proteins (Fig. 1p). Hence, mature neurons express the full enzymatic machinery necessary to oxidize long-chain fatty acids for mitochondrial ATP production.

**Extended Data Fig. 3:**
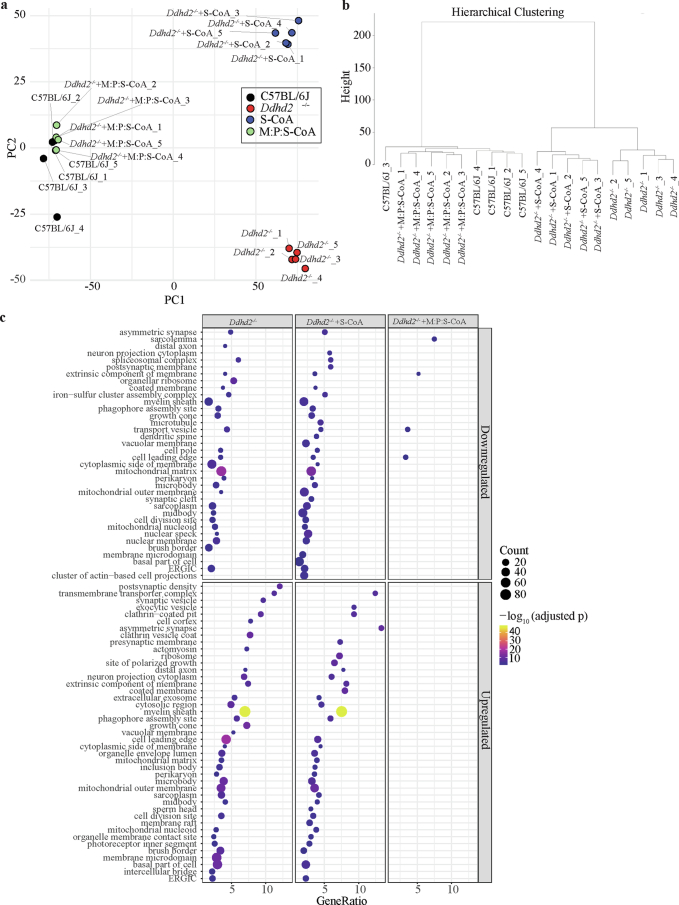
Mass spectrometry analysis of neuronal cultures of C57BL/6J, *Ddhd2*^*−/−*^ and *Ddhd2*^*−/−*^ treated with fatty acyl-CoA supplements. Global proteomic shifts and rescue effects in *Ddhd2*^*−/−*^ neurons revealed by PCA, hierarchical clustering, and GO enrichment. **a**, Principal component analysis (PCA) of batch-corrected protein abundance data from C57BL/6J, *Ddhd2*^*−/−*^, and *Ddhd2*^*−/−*^ neurons treated with either S-CoA or M:P:S-CoA. Each point represents an individual sample, with the outer square indicating replicate identity and inner fill denoting treatment group. PC1 and PC2 account for the greatest variance in the dataset and show clear separation by condition. **b**, Hierarchical clustering based on Euclidean distance and complete linkage, using normalized and batch-corrected protein abundance data. Samples cluster by experimental group, with M:P:S-CoA-treated *Ddhd2*^*−/−*^ neurons displaying the closest proximity to wild-type samples. **c**, Gene Ontology (GO) enrichment analysis (cellular component category) of significantly up- or down-regulated proteins in *Ddhd2*^*−/−*^ ± S-CoA or M:P:S-CoA relative to C57BL/6J. Dot size represents the number of enriched proteins; color scale reflects statistical significance (adjusted p-value); and GeneRatio corresponds to the fraction of significant proteins per GO term. N = 5 biologically independent experiments in each condition.

To assess whether the abundance of β-oxidation and carnitine cycle proteins was altered by the loss of Ddhd2, LFQ LC–MS/MS proteomics was performed on C57BL/6J and *Ddhd2*^−/−^ neurons. *Ddhd2*^−/−^ neurons showed widespread changes in fatty acid metabolism proteins, including significant upregulation of Cpt1a compared to controls (Fig. 1q). Replicate reproducibility was confirmed by principal component analysis (Extended Data Fig. 3a) and hierarchical clustering (Extended Data Fig. 3b), confirming that the proteome identified in each replicate consistently clustered according to its respective experimental group (that is, C57BL/6J, *Ddhd2*^−/−^ ± S-CoA or M:P:S-CoA). The alterations in the proteostasis were partially corrected by 48-h 1 µM S-CoA treatment and more effectively by combined 1 µM M:P:S-CoA supplementation over the same treatment period (Fig. 1q and Extended Data Fig. 3c). These findings suggest that M-CoA, P-CoA and S-CoA may also support other neuronal functions beyond energy fuelling. For instance, myristic and palmitic acids have well established roles in protein *N*-myristoylation and S-palmitoylation, respectively, which affect the stability, subcellular localization and membrane anchoring of various proteins, thereby affecting a multitude of cellular pathways^27–31^.

### Metabolic crosstalk between β-oxidation and glycolysis

**Extended Data Fig. 4:**
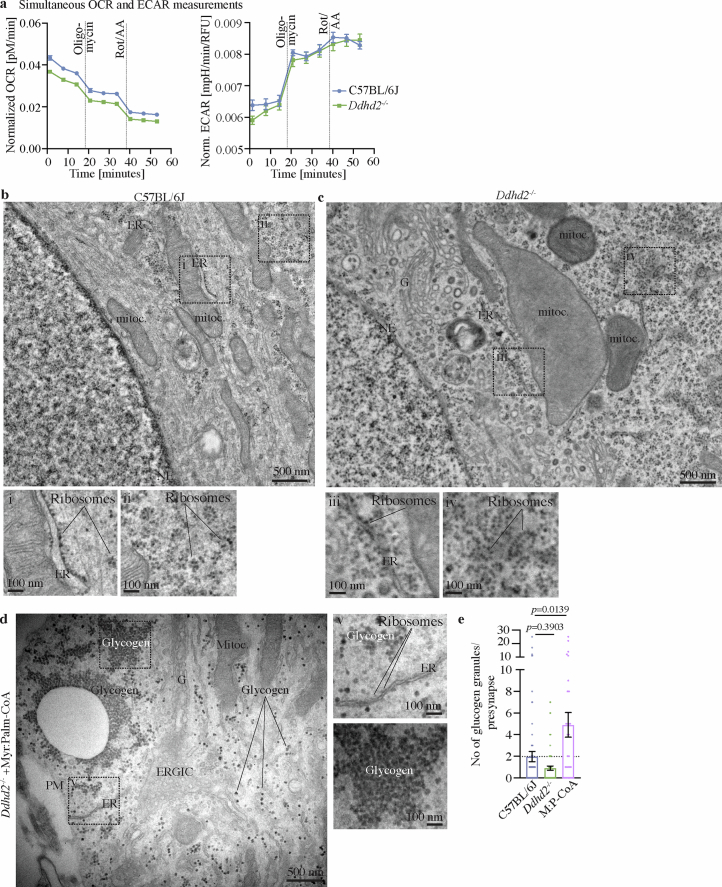
Inhibition of mitochondrial respiration increases ECAR while external supplementation of fatty acyl-CoA leads to an increase in glycogen. **a**, Simultaneous OCR and ECAR measurements in neuronal C57BL/6J and *Ddhd2*^*−/−*^cultures. Injection of oligomycin, and Rot/AA are indicated. Representative EM images of E16 neuronal cultures ( + 4 µM Ara-C) of **b**, C57BL/6J, **c**, *Ddhd2*^*−/−*^ and **d**, *Ddhd2*^*−/−*^ + 1 µM M:P-CoA for 48 h and imaged at DIV21. Boxed areas (i-iv) are shown magnified. Endoplasmic reticulum (ER), mitochondria (mitoc.), nuclear envelope (NE), Golgi complex (G), ribosomes, glycogen and plasma membrane (PM) are indicated. Note the size difference between ribosomes and glycogen for identification. **e**, EM quantification of average number of glycogen granules in synapses in C57BL/6J, and *Ddhd2*^*−/−*^ ± 1 µM M:P-CoA for 48 h. Data are presented as mean values ± SEM, dots present quantifications in synapses from technical replicates. N = 3 (**a, e**,) biologically independent experiments in each condition. The exact *p*-values stated in the graphs were determined from biological replicates using one-way ANOVA Kruskal-Wallis multiple comparisons test (**e**,).

Building on the finding that *Ddhd2*^−/−^ neurons exhibit increased glycolysis and reduced mitochondrial respiration, we further examined the interplay between these metabolic pathways. Simultaneous OCR (respiration) and ECAR (glycolysis) measurements confirmed reduced OCR in *Ddhd2*^−/−^ neurons (Extended Data Fig. 4a). To test whether blocking mitochondrial ATP synthesis would upregulate glycolysis, we treated neurons with oligomycin, an ATP synthase inhibitor, and rotenone and antimycin A (Rot/AA), which block the electron transport chain. Following these treatments, ECAR increased in both neuron types, indicating enhanced glycolytic compensation (Extended Data Fig. 4a).

**Fig. 2:**
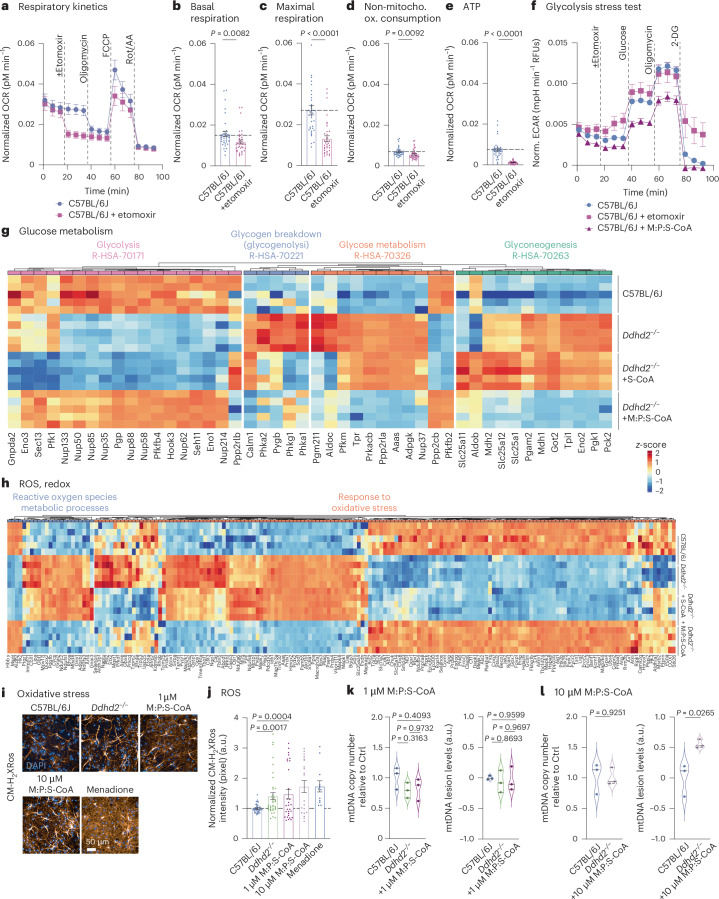
Mitochondrial fatty acid import regulates neuronal energy balance without inducing ROS or mtDNA damage. **a**–**e**, Seahorse XF OCR kinetics (**a**) and quantification of basal respiration (**b**), maximal respiration (**c**), non-mitochondrial oxygen consumption (**d**) and ATP production (**e**) in E16 C57BL/6J hippocampal neuronal cultures ± etomoxir at DIV 21–22. Injection of etomoxir, oligomycin, FCCP and Rot/AA is indicated. **f**, Seahorse XF ECAR glycolysis stress test kinetics in DIV 21–22 hippocampal neuronal cultures of E16 C57BL/6J treated with etomoxir or 1 µM M:P:S-CoA for 48 h. Injection of etomoxir, glucose, oligomycin and 2-DG is indicated. **g**, LFQ LC–MS/MS protein abundance analysis of gluconeogenesis (R-HSA-70263), glucose metabolism (R-HSA-70326), glucose breakdown (gluconeogenesis, R-HSA-70221) and glycolysis (R-HAS-70171) proteins at DIV 21–22 cortical neuron cultures of E16 C57BL/6J, *Ddhd2*^−/−^ ± 1 µM S-CoA or 1 µM M:P:S-CoA treatment for 48 h, showing column-wise *z*-score-normalized protein abundances in each condition. **h**, LFQ LC–MS/MS analysis of oxidative stress (GO:0006979) and ROS metabolic process protein abundance at DIV 21–22 in E16 cortical neuron cultures of C57BL/6J, and *Ddhd2*^−/−^ ± 1 µM S-CoA or 1 µM M:P:S-CoA for 48 h, showing column-wise *z*-score-normalized protein abundances in each condition. **i**,**j**, Representative fluorescence images (**i**) and quantification (**j**) of CMHrXRos fluorescence intensity in E16 hippocampal neuron cultures of C57BL/6J and *Ddhd2*^−/−^ ± 1 µM or 10 µM M:P:S-CoA for 48 h, or menadione. **k**,**l**, qPCR analysis of mtDNA copy number and mtDNA lesion levels in cortical neuron cultures of C57BL/6J, and *Ddhd2*^−/−^ treated with 1 µM M:P:S-CoA (**k**) or 10 µM M:P:S-CoA (**l**) for 48 h. Proteomics samples and proteins were clustered using Euclidean distance and complete linkage, and the colour gradients in heat maps reflect *z*-scores from low (blue) to high (red) abundance, each column representing a unique protein and each row a biological replicate. Data are presented as mean values ± s.e.m.; dots present averages of biological replicates. Sample sizes are *N* = 3 (**a**–**f**, **k** and **l**) and *N* = 5 (**g**, **h**, and C57BL/6J, *Ddhd2*^−/−^ and *Ddhd2*^−/−^ ± 1 µM M:P:S-CoA in **j**), *N* = 2 (*Ddhd2*^−/−^ ± 1 µM M:P:S-CoA and *Ddhd2*^−/−^ + menadione in **j**) biologically independent experiments in each condition. The exact *P* values stated in the graphs were determined from biological replicates using a two-tailed unpaired *t*-test (**b**, **d**, **e** and **l**), two-tailed Mann–Whitney test (**c**), and ordinary one-way ANOVA Kruskal–Wallis multiple-comparisons test (**j**) and ordinary one-way ANOVA Tukey’s multiple-comparisons test (**k**).

To investigate mitochondrial fatty acid import inhibition in wild-type neurons, C57BL/6J neurons were treated with etomoxir, and the OCR was measured. Etomoxir treatment reduced mitochondrial respiration and ATP production significantly compared to vehicle-treated controls (Fig. 2a–e). The decrease in ATP levels in etomoxir-treated C57BL/6J neurons was more drastic compared to *Ddhd2*^−/−^ neurons (Fig. 1k), which show decreased but not depleted sFFA levels^3^. Acute Cpt1 inhibition also caused significantly lowered maximal respiration, a hallmark of mitochondrial dysfunction often seen in neurodegenerative diseases^32^, similarly to our observations in *Ddhd2*^−/−^ neurons (Fig. 1i). Additionally, non-mitochondrial OCRs decreased significantly following etomoxir treatment (Fig. 2d), and a similar trend was also observed in *Ddhd2*^−/−^ neurons (Fig. 1j), suggesting that the stress from perturbed FFA import and energy production may suppress broader cellular metabolism. Together, these findings demonstrate that neurons can oxidize both endogenous and exogenous CoA-activated fatty acids and that, similarly to non-neuronal cells, Cpt1-mediated mitochondrial import of long-chain fatty acids is essential for β-oxidation and energy production.

To further assess how acute β-oxidation inhibition affects glycolysis, ECAR was measured in etomoxir-treated C57BL/6J neurons. Etomoxir elevated ECAR (Fig. 2f), mirroring the increase observed in *Ddhd2*^−/−^ neurons (Fig. 1a–e). In contrast, a 48-h M:P:S-CoA treatment reduced ECAR in C57BL/6J neurons (Fig. 2f). Fatty acyl-CoA supplementation also increased glycogen storage in *Ddhd2*^−/−^ neurons (Extended Data Fig. 4b–e).

We next examined the impact of Ddhd2 loss on proteins involved in glucose metabolism. LFQ LC–MS/MS analysis showed a general decrease in glycolytic proteins and an increase in proteins associated with glycogenolysis, gluconeogenesis and broader glucose metabolism in *Ddhd2*^−/−^ neurons compared to controls (Fig. 2g). While 1 µM S-CoA partially restored these changes, combined 1 µM M:P:S-CoA treatment normalized proteostasis to control levels (Fig. 2g). These findings suggest an inverse metabolic cross-talk: when ATP levels are restored via β-oxidation, glycolysis slows, and excess glucose is stored as glycogen and when FFA levels drop, glucose stores are mobilized to meet energy demands.

Because β-oxidation can elevate reactive oxygen species (ROS) and contribute to oxidative stress, we assessed whether fatty acyl-CoA supplementation increased ROS in neurons. Previous studies linked Ddhd2 loss to elevated ROS in mouse embryonic fibroblasts^12^. Consistent with this, LFQ LC–MS/MS revealed increased expression of oxidative stress-related proteins in *Ddhd2*^−/−^ neurons, which was not further exacerbated by the M:P:S-CoA treatment (Fig. 2h). MitoTracker Red CM-H2XRos, a reduced dye that remains sequestered to mitochondria once it is oxidized by ROS, was used to visualize ROS (Fig. 2i). Quantification of CM-H2XRos intensity confirmed significantly elevated ROS levels in *Ddhd2*^−/−^ neurons compared to C57BL/6J controls, consistent with prior findings in mouse embryonic fibroblasts^18^ (Fig. 2j). Notably, treatment with 1 µM M:P:S-CoA for 48 h did not increase ROS levels in *Ddhd2*^−/−^ neurons, whereas a higher dose (10 µM) showed a trend of elevated ROS (Fig. 2j). Menadione-treated *Ddhd2*^−/−^ neurons served as a positive control for ROS induction. As elevated ROS can damage mitochondrial DNA (mtDNA), mtDNA integrity was assessed in C57BL/6J and *Ddhd2*^−/−^ neurons treated with 1 µM or 10 µM M:P:S-CoA for 48 h using qPCR. We observed no damages in mtDNA in *Ddhd2*^*−/−*^ neurons or following 1 µM M:P:S-CoA treatment (Fig. 2k). However, 10 µM M:P:S-CoA significantly increased mtDNA lesions, without affecting mtDNA copy number (Fig. 2l). These findings highlight the need to carefully optimize M:P:S-CoA dosing for potential therapeutic use to avoid oxidative stress and mtDNA damage.

### Exogenous fatty acyl-CoA supplementation rescues altered mitochondrial structure and localization in *Ddhd2*^−/−^ neurons

**Fig. 3:**
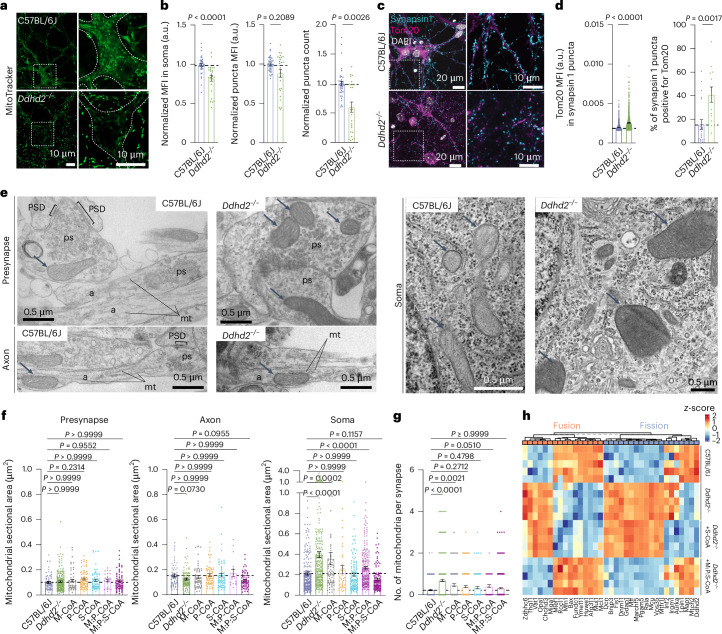
Defects in mitochondrial structure, subcellular localization and ATP production in *Ddhd2*^−/−^ neurons are rescued with combined fatty acyl-CoA supplements. **a**, Representative images of MitoTracker green staining in live E16 C57BL/6J and *Ddhd2*^−/−^ hippocampal neurons at DIV 21–22. Boxed areas are magnified in the bottom showing neuronal soma outlines and a decrease in fluorescence in *Ddhd2*^−/−^ neuron soma. See also Supplementary Videos 1 and 2. **b**, Quantifications of MitoTracker Deep Red FM staining in E16 C57BL/6J and *Ddhd2*^−/−^ hippocampal neurons imaged with high-content live imaging at DIV 21–22 showing MFI (a.u.) in the neuronal soma normalized to C57BL/6J, MFI of MitoTracker-stained puncta normalized to puncta background, and MitoTracker puncta count normalized to puncta background per cell (DAPI). **c**, Representative immunofluorescence images of endogenous Tom20 and synapsin 1 immunostaining in E16 C57BL/6J and *Ddhd2*^−/−^ hippocampal neurons at DIV 21–22. DAPI staining shown for reference. **d**, Quantifications show Tom20 MFI (a.u.) in synapsin 1 puncta and the percentage of synapsin 1 puncta positive for Tom20. The dotted horizontal lines indicate the average of the C57BL/6J controls. **e**, Representative electron microscopy images of E16 C57BL/6J and *Ddhd2*^−/−^ hippocampal neuron cultures at DIV 21–22 showing presynapses (ps), axons (a), soma, microtubules (mt), postsynaptic density (PSD), nuclear envelope (NE), endoplasmic reticulum (E), Golgi complex (G) and mitochondria (arrows). **f**,**g**, Quantification of mitochondrial sectional area (µm^2^) from electron microscopy images (**f**) and the number of mitochondrial profiles per presynapse (**g**) in indicated conditions. **h**, LFQ LC–MS/MS analysis of mitochondrial fusion (GO:0008053) and fission (GO:0000266) protein abundance at DIV 21–22 in cortical neuron cultures of E16 C57BL/6J, and *Ddhd2*^−/−^ ± 1 µM S-CoA or 1 µM M:P:S-CoA for 48 h, showing column-wise *z*-score-normalized abundances of pathways. Proteomics samples and proteins were clustered using Euclidean distance and complete linkage, and the colour gradients in heat maps reflect *z*-scores from low (blue) to high (red) abundance, each column representing a unique protein and each row a biological replicate. Data are presented as mean values ± s.e.m.; dots indicate technical replicates (**b**) and individual quantifications of synapses from technical replicates (**d**, **f** and **g**). The exact *P* values stated in the graphs were determined from biological replicates using a two-tailed Mann–Whitney test (1st graph in **b** and **c**) and a two-tailed unpaired *t*-test (2nd and 3rd graphs in **b** and 2nd graph in **c**) and one-way ANOVA Kruskal–Wallis multiple-comparison test (**f** and **g**). Sample sizes were *N* = 3 (**b**, **d**, **f** and **g**) and *N* = 5 (**h**) biologically independent experiments in each condition.

To further examine mitochondrial changes following Ddhd2 loss, C57BL/6J and *Ddhd2*^−/−^ neurons were stained with MitoTracker Green FM and imaged live by confocal microscopy. *Ddhd2*^−/−^ neurons showed reduced perinuclear and somatic MitoTracker intensity compared to controls (Fig. 3a and Supplementary Videos 1 and 2). High-content live-cell imaging and automated analysis, performed as previously described^33^, confirmed significantly lower mean fluorescence intensity (MFI) of MitoTracker Deep Red FM in the perinuclear region, along with a decrease in overall MFI and significantly fewer MitoTracker-stained puncta in *Ddhd2*^−^^/^^−^ neurons compared to controls (Fig. 3b), suggesting a reduction in active mitochondria. Immunostaining for mitochondrial protein Tom20 and synaptic marker synapsin 1 revealed altered patterns (Fig. 3c). Quantification showed a significantly increased Tom20 MFI within synapsin-1-positive puncta and higher percentage of synapsin 1 puncta positive for Tom20 in *Ddhd2*^−/−^ neuron synapses (Fig. 3d). Electron microscopy confirmed these findings, revealing irregular and enlarged somatic mitochondria in *Ddhd2*^−/−^ neurons compared to C57BL/6J controls, with no apparent changes in mitochondrial size or morphology in axons or presynapses (Fig. 3e). Quantification of mitochondrial area confirmed significant enlargement in the soma but not in axons or synapses in *Ddhd2*^−^^/^^−^ neurons compared to controls (Fig. 3f). A 48-h treatment with 1 µM fatty acyl-CoA supplements, especially when combined, restored somatic mitochondrial size to control levels (Fig. 3f). Consistent with the observed increase in Tom20 localization in synapses (Fig. 3c,d), electron microscopy analysis also showed significantly increased number of presynaptic mitochondria in *Ddhd2*^−/−^ neurons compared to controls, likely as a compensatory response, which was reversed by fatty acyl-CoA supplementation (Fig. 3g). Supporting these structural findings, LFQ LC–MS/MS proteomics revealed widespread alterations in mitochondrial fusion and fission proteins in *Ddhd2*^−/−^ neurons compared to C57BL/6J controls (Fig. 3h). A 48-h 1 µM S-CoA treatment partially corrected these changes, while combined 1 µM M:P:S-CoA treatment restored protein levels to control levels (Fig. 3h). Together, these results indicate that Ddhd2 loss alters mitochondrial structure and localization, leading to larger mitochondria accumulation in the soma and an increased presence of mitochondria in synapses.

### Synaptic energy loss from Ddhd2 deficiency is rescued by fatty acyl-CoA supplementation

**Fig. 4:**
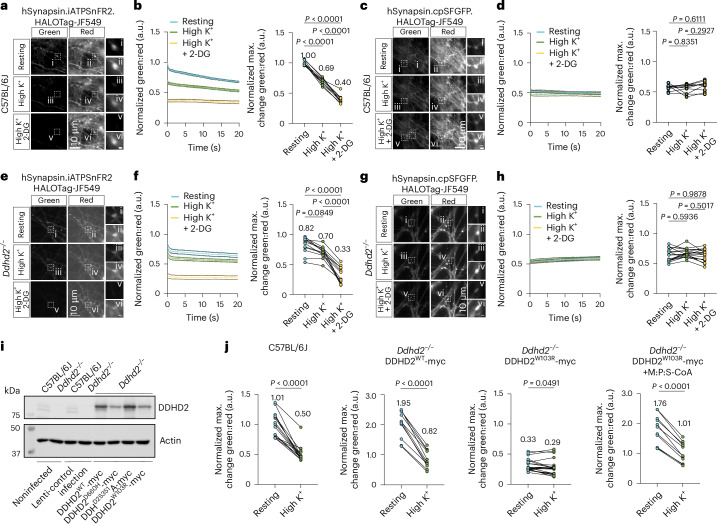
Synaptic energy defects observed in HSP54-associated *DDHD2* mutation are rescued with combined fatty acyl-CoA supplements. **a**, Representative, simultaneously acquired, fluorescence images of green (iATPSnFR2) and red (HaloTag-Janelia Fluor 549) fluorescence of ratio-metric hSynapsin.iATPSnFR2.HaloTag-JF549 ATP sensor at rest, during high K^+^ stimulation, and following a 15-min high K^+^ stimulation in the presence of 2-DG in E16 cultured hippocampal C57BL/6J neurons at DIV18-19. Boxed areas (i–vi) are shown magnified on the right. **b**, Kinetic graph shows the average ratio (normalized average numbers indicated on top of each condition) of iATPSnFR2 green to HaloTag-Janelia Fluor 549 red fluorescence and maximum change in green:red normalized to the C57BL/6J/ATP sensor at *t*_1_. **c**, Representative, simultaneously acquired, fluorescence images of green (cpSFGFP) and red (HaloTag-Janelia Fluor 549) fluorescence of ratio-metric hSynapsin.cpSFGFP.HaloTag-JF549 non-responsive control at rest, during high K^+^ stimulation, and following a 15-min high K^+^ stimulation in the presence of 2-DG in E16 cultured hippocampal C57BL/6J neurons at DIV18-19. Boxed areas (i–vi) are shown magnified on the right. **d**, Kinetic graph shows the average ratio (normalized average numbers indicated on top of each condition) of cpSFGFP green to HaloTag-Janelia Fluor 549 red fluorescence and maximum change in green:red normalized to the C57BL/6J/ATP sensor at *t*_1_. **e**, Representative, simultaneously acquired, fluorescence images of green (iATPSnFR2) and red (HaloTag-Janelia Fluor 549) fluorescence of ratio-metric hSynapsin.iATPSnFR2.HaloTag-JF549 ATP sensor at rest, during high K^+^ stimulation, and following a 15-min high K^+^ stimulation in the presence of 2-DG in E16 cultured hippocampal *Ddhd2*^−^^/^^−^ neurons at DIV18-19. Boxed areas (i–vi) are shown magnified on the right. **f**, Kinetic graph shows the average ratio (normalized average numbers indicated on top of each condition) of iATPSnFR2 green to HaloTag-Janelia Fluor 549 red fluorescence and maximum change in green:red normalized to the C57BL/6J/ATP sensor at *t*_1_. **g**, Representative, simultaneously acquired, fluorescence images of green (cpSFGFP) and red (HaloTag-Janelia Fluor 549) fluorescence of ratio-metric hSynapsin.cpSFGFP.HaloTag-JF549 non-responsive control at rest, during high K^+^ stimulation, and following a 15-min high K^+^ stimulation in the presence of 2-DG in E16 cultured hippocampal *Ddhd2*^−^^/^^−^ neurons at DIV18-19. Boxed areas (i–vi) are shown magnified on the right. **h**, Kinetic graph shows the average ratio (normalized average numbers indicated on top of each condition) of cpSFGFP green to HaloTag-Janelia Fluor 549 red fluorescence and maximum change in green:red normalized to the C57BL/6J/ATP sensor at *t*_1_. **i**, Western blot of DDHD2 protein levels in C57BL/6J and *Ddhd2*^−^^/^^−^ ± lenti control infection, DDHD2^wt^-myc, DDHD2^D660H^-myc, DDHD2^S351A^-myc and DDHD2^W103R^-myc. **j**, Hippocampal C57BL/6J neurons, and lentivirally induced *Ddhd2*^−^^/^^−^ hippocampal neurons expressing DDHD2^wt^-myc and DDHD2^W103R^-myc, were AAV9-infected to express hSynapsin.iATPSnFR2.HaloTag-JF549. Quantification of the hSynapsin.iATPSnFR2.HaloTag-JF549 maximum change in green:red normalized to the C57BL/6J/ATP sensor at *t*_1_ in E16 hippocampal C57BL/6J, and *Ddhd2*^−^^/^^−^ neurons transiently expressing DDHD2^wt^-myc and DDHD2^W103R^-myc ± 1 µM M:P:S-CoA for 48 h at DIV14-16. Data are presented as mean values ± SEM from individual synapses. The exact *P* values stated in the graphs were determined from biological replicates using one-way ANOVA Kruskal–Wallis multiple comparison test (**b**), one-way ANOVA Tukey’s multiple comparisons test (**f**, **d** and **h**) and two-tailed paired *t*-test (**j**). Sample sizes were *N* = 3 in **a** and **b**, and *Ddhd2*^−^^/^^−^ +DDHD2^wt^-myc and *Ddhd2*^−^^/^^−^ +DDHD2^W103R^-myc + M:P:S-CoA in **j**, and *N* = 4 in **d**, **f** and **h**, and C57BL/6J, *Ddhd2*^−^^/^^−^ +DDHD2^W103R^-myc in **j**, biologically independent experiments. Source data

Synaptic ATP levels were next assessed using a genetically encoded ATP sensor enabling real-time ratio-metric measurement of ATP dynamics at the synapse^34^. Adeno-associated virus serotype 9 (AAV9)-mediated expression of hSynapsin.iATPSnFR2.HALOTag^34^ (ATP sensor) or the nonresponsive control sensor hSynapsin.cpSFGFP.HALOTag^34^ was introduced into cultured C57BL/6J and *Ddhd2*^−/−^ neurons at DIV 9. The sensor emits green fluorescence proportional to ATP levels, and constant red fluorescence after binding to an exogenously provided chemical ligand (membrane-permeable Janelia Fluor 549 HaloTag ligand, JF549), which is used as a normalization control to account for the expression levels of the construct. At DIV 18–19, neurons were labelled with HaloTag ligand JF549 and imaged using total internal reflection fluorescence (TIRF) live-cell imaging through simultaneous green/red recording. Synaptic ATP levels were estimated as the green:red fluorescence ratio at rest, after high K⁺ stimulation, and following glycolysis inhibition with 2-deoxy-d-glucose (2-DG) under stimulatory conditions. In C57BL/6J neurons, high K⁺ stimulation decreased the green:red ratio by 31%, and 2-DG further reduced it by 43% (Fig. 4a,b), with no changes seen in neurons expressing the nonresponsive control (Fig. 4c,d), confirming the sensitivity and specificity of the ATP sensor^34^. In *Ddhd2*^−/−^ neurons, baseline synaptic ATP was 18% lower than controls (Fig. 4e,f) and decreased only modestly (14%) with stimulation but dropped a further 53% after 2-DG treatment. No changes were observed with the inactive control (Fig. 4g,h). These results demonstrated that despite the increased presence of mitochondria in *Ddhd2*^−/−^ synapses, the ATP levels were reduced at basal conditions and failed to respond robustly to stimulation. Comparatively, the decreased ATP levels following the strong high K^+^ stimulation in wild-type neurons approximated the basal ATP levels in Ddhd2-deficient neurons.

**Extended data Fig. 5:**
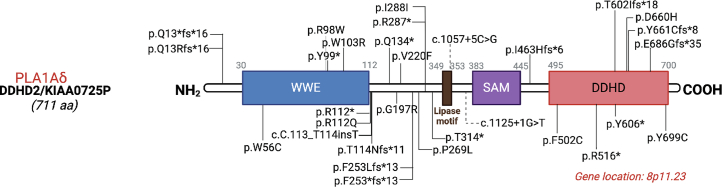
Reported human mutations of *DDHD2* gene. See Table 4 for further details. Created in BioRender.com.

To determine whether fatty acyl-CoA supplementation could reverse HSP54-associated disease states in neurons, the effects of human *DDHD2* mutations were examined. Multiple HSP54-linked *DDHD2* mutations have been reported^6,11,13–15,35–46^ (Extended Data Fig. 5a and Supplementary Table 3). Among these, the p.Trp103Arg (W103R) mutation, predicted to disrupt the DDHD and WWE domains, has been linked to childhood-onset HSP54 characterized by intellectual disability and developmental delay^41^. To model this mutation, lentiviral constructs expressing Myc-tagged DDHD2 wild-type (DDHD2^WT^-myc) and W103R^12^ (DDHD2^W103R^-myc) were generated. For comparison, another HSP54-associated mutant p.Asp660His^41^ (DDHD2^D660H^-myc), and a catalytically inactive p.Ser351Ala mutant^1,12^ (DDHD2^S351A^-myc), were also introduced into *Ddhd2*^−/−^ neuron–glia cultures at DIV 3 using a multiplicity of infection (MOI) of 1. Western blot analysis at DIV 14 confirmed that DDHD2^D660H^ and DDHD2^W103R^ mutants were less stable than DDHD2^WT^ or DDHD2^S351A^ (Fig. 4i), consistent with reduced protein levels observed in individuals with HSP54. The disease mutant W103R was selected for further functional analysis. At DIV 3, *Ddhd2*^−/−^ neuron–glia cultures were transduced with lentiviral vectors expressing DDHD2^WT^ or DDHD2^W103R^ (MOI 10), followed by AAV9-delivered ATP sensors (MOI 1) at DIV 9. Synaptic ATP levels were monitored by green/red ratio-metric imaging at DIV 14–16. Compared to C57BL/6J neurons, DDHD2^WT^ overexpression doubled synaptic ATP levels, while DDHD2^W103R^ overexpression resulted in a 70% reduction (Fig. 4j) in *Ddhd2*^−/−^ neurons. A 48-h treatment with 1 µM M:P:S-CoA increased synaptic ATP by 80% in resting DDHD2^W103R^-expressing neurons, and restored the responsiveness to high K^+^ stimulation (Fig. 4j), demonstrating that fatty acyl-CoA supplementation can rescue the bioenergetic deficits associated with HSP54.

### *Ddhd2*^−/−^ neurons have an altered proteome that is largely rebalanced by M:P:S-CoA supplementation

**Fig. 5:**
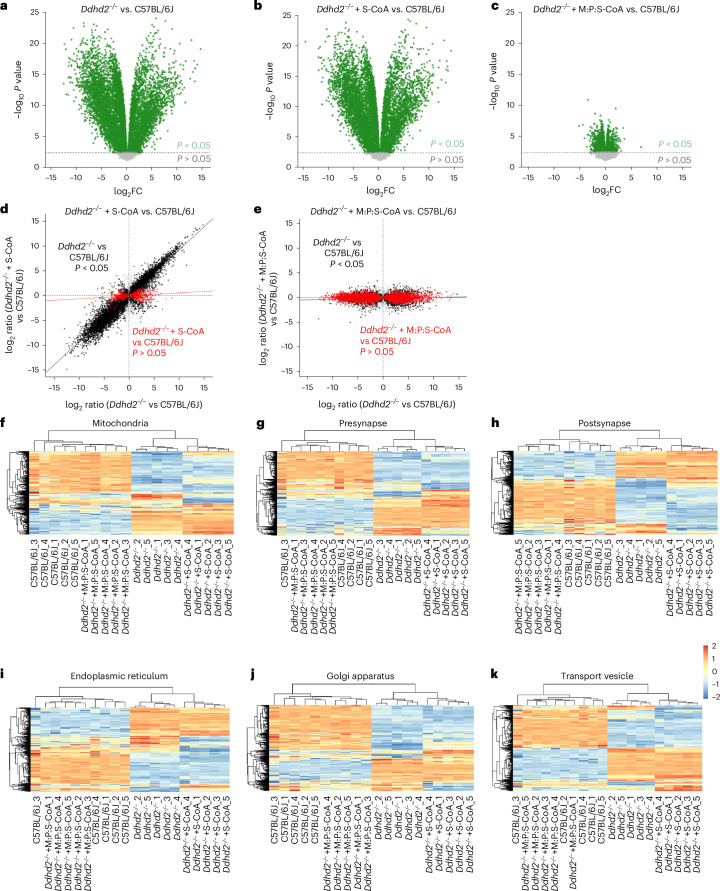
*Ddhd2*^−/−^ neuron proteome imbalance is restored with a combined fatty acyl-CoA treatment. **a**–**c**, Volcano plots of LFQ LC–MS/MS global proteomics comparing cultured E16 cortical C57BL/6J and *Ddhd2*^−/−^ neurons across the indicated experimental conditions at DIV 21–22 showing *Ddhd2*^−/−^ versus C57BL/6J (**a**), *Ddhd2*^−/−^ + 1 µM S-CoA for 48 h versus C57BL/6J (**b**) and *Ddhd2*^*−/−*^ + 1 µM M:P:S-CoA for 48 h versus C57BL/6J (**c**). Differentially abundant proteins are displayed as log_2_ fold change (*x* axis) and −log_10_-transformed *P* values (*y* axis). The significance threshold (*P* < 0.05) is indicated by a dashed horizontal line. Proteins with *P* < 0.05 are shown in green; non-significant proteins are shown in grey. Rescue dot plots display protein abundance changes in *Ddhd2*^−/−^ versus C57BL/6J (*x* axis) and rescue with S-CoA (**d**) and M:P:S-CoA (**e**) versus C57BL/6J (*y* axis). Each dot represents a protein; proteins with significant and nonsignificant differences in abundance in *Ddhd2*^−/−^ compared to C57BL/6J are shown in black and grey, respectively. Proteins, the abundance of which following treatments no longer significantly differed from the C57BL/6J controls, are shown in red. Red and black lines indicate linear model fits for rescued and still dysregulated protein subsets, respectively, visualizing proteome convergence towards wild-type (C57BL/6J) levels. **f**–**k**, Heat maps displaying row-wise *z*-score-normalized protein abundances across six functional compartments: mitochondria (GO:0005739) (**f**), presynapse (GO:0098793) (**g**), postsynapse (GO:0098794) (**h**), endoplasmic reticulum (GO:0005783) (**i**), Golgi apparatus (GO:0005794) (**j**) and transport vesicle (GO:0030133) (**k**). Gene sets were selected from enriched GO terms specific to each compartment. Each row represents a unique protein, and each column represents a biological replicate. Samples and proteins were clustered using Euclidean distance and complete linkage. Colour gradients reflect *z*-scores from low (blue) to high (red) abundance. *N* = 5 biologically independent cell culture preparations in each condition.

**Extended Data Fig. 6:**
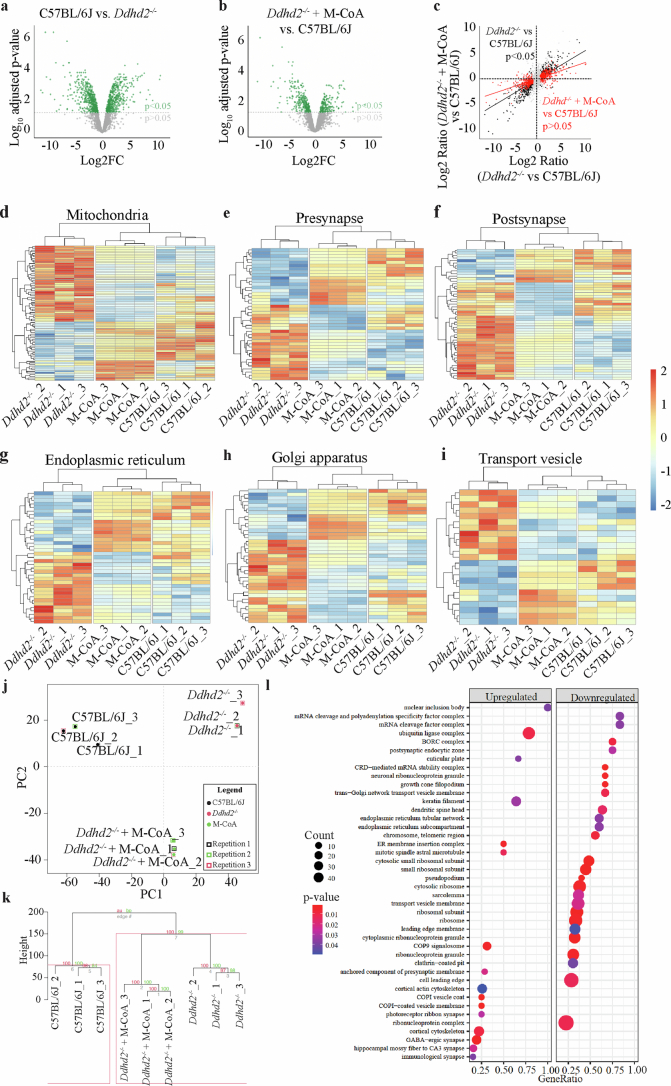
Global proteomics in C57BL/6J, and *Ddhd2*^*−/−*^ ± M-CoA, neuron-glia cultures. Volcano plot of mass spectrometry proteomics of cultured neuron-glia cultures (no Ara-C) of E16 **a**, C57BL/6J vs. *Ddhd2*^*−/−*^ and **b**, *Ddhd2*^*−/−*^ treated with 1 µM M-CoA for 48 h vs. C57BL/6J. Altered protein abundance is represented as the log_2_ FC (or ratio) of the C57BL/6J vs. *Ddhd2*^*−/−*^ (**a**, X-axis) and *Ddhd2*^*−/−*^ + M-CoA vs. C57BL/6J (**b**, X-axis), and -log_10_ transformed adjusted p-value (Y-axes, p < 0.05 threshold indicated as a dotted grey line). **c**, Protein abundance of *Ddhd2*^*−/−*^ vs. C57BL/6J (X-axis) and *Ddhd2*^*−/−*^ + M-CoA vs. C57BL/6J (Y-axis). Proteins with unchanged levels (grey dots) and those significantly altered in abundance in *Ddhd2*^*−/−*^ vs C57BL/6J (965 proteins, black dots) are highlighted. Notably, 605 proteins (red dots) regain normal levels after M-CoA rescue in *DDHD2*^*−/−*^. The black and red lines correspond to linear model fits to these sets of proteins, highlighting the *Ddhd2*^*−/−*^ proteome shift towards C57BL/6J. **d-i** Protein abundance heatmaps for targeted pathways for mitochondria (GO:0005739), presynapse (GO:0098793), postsynapse’ (GO:XXX), endoplasmic reticulum (GO:0005783), Golgi apparatus (GO:0005794), and transport vesicle (GO:0030133). Each row represents an individual protein. Cells are coloured by the z-score calculated for each row. Samples (columns) and proteins (rows) are clustered Euclidean method with complete linkage. **j**, Principal component analysis of neuron-glia co-cultures of E16 C57BL/6J, *Ddhd2*^*−/−*^ and *Ddhd2*^*−/−*^ treated with 1 µM M-CoA for 48 h and analysed with mass spectrometry at DIV21-22. **k**, Hierarchical clustering. Samples cluster according to their experimental group (within each of the tree-like - dendrograms). Values on branches and the red box, represent probabilities of observing the clustering after 100 bootstraps. **l**, Gene set enrichment analysis (GSEA). Top Cellular component terms associated with increased abundance (upregulated) or decreased abundance (downregulated) upon M-CoA treatment in *Ddhd2*^*−/−*^ neurons are shown. Count, also the size of the point, is the number of proteins associated with increased or decreased abundance. GeneRatio is the proportion of proteins captured as increased or decreased in abundance. P-value, also the colour of each point, is the significance of enrichment of proteins associated with each cellular component term. N = 3 biologically independent experiments in each condition.

To characterize the cellular impact of Ddhd2 loss, we performed LFQ LC–MS/MS on cultured C57BL/6J and *Ddhd2*^−/−^ neurons and assessed the effects of 48-h supplementation with 1 µM S-CoA or M:P:S-CoA. Principal component analysis and hierarchical clustering confirmed replicate reproducibility (Extended Data Fig. 3a,b). In total, 9,111 proteins were identified across five biological replicates (Supplementary Table 4). *Ddhd2*^−/−^ neurons exhibited widespread proteomic shifts, with 3,014 proteins upregulated and 5,014 downregulated (*P* < 0.05; Fig. 5a). S-CoA treatment had only a modest restoration effect (2,852 proteins upregulated and 5,176 downregulated; Fig. 5b), whereas M:P:S-CoA markedly reduced the number of altered proteins in *Ddhd2*^−/−^ neurons (1,050 and 1,484, respectively; Fig. 5c) compared to controls. Rescue analysis showed that S-CoA corrected only 8.4% of dysregulated proteins (Fig. 5d), while M:P:S-CoA restored 72.3% (Fig. 5e). Gene Ontology (GO) analysis revealed broad disruptions in mitochondrial, synaptic, endoplasmic reticulum, Golgi and vesicle-associated proteins (Fig. 5f–k), which were substantially reversed by M:P:S-CoA but not S-CoA (Extended Data Fig. 3c) treatments. Treatment with M-CoA was sufficient to rescue over 60% of the 965 significantly altered proteins and restoring pathway profiles towards controls in *Ddhd2*^−/−^ neuron–glia cultures (Extended Data Fig. 6 and Supplementary Table 5).

Together with earlier results showing mitochondrial ATP rescue with 48-h treatment (Fig. 1g–k), as well as an incremental proteostasis restoration by M:P:S-CoA > M-CoA > S-CoA, these results indicate that recovery of proteostasis is not solely dependent on restoring energy metabolism. Rather, they suggest that M-CoA and P-CoA contribute additional functional roles (for example, restoring protein lipidation), which is a time-dependent process. Overall, these findings demonstrate that the widespread proteomic imbalances observed in Ddhd2-deficient neurons and neuron–glia cultures are largely reversible, and that a 48-h treatment with M:P:S-CoA is sufficient to substantially re-establish proteostasis to a control-like state.

### Fatty acyl-CoA supplementation restores protein trafficking in the secretory pathway of *Ddhd2*^−/−^ neurons

**Fig. 6:**
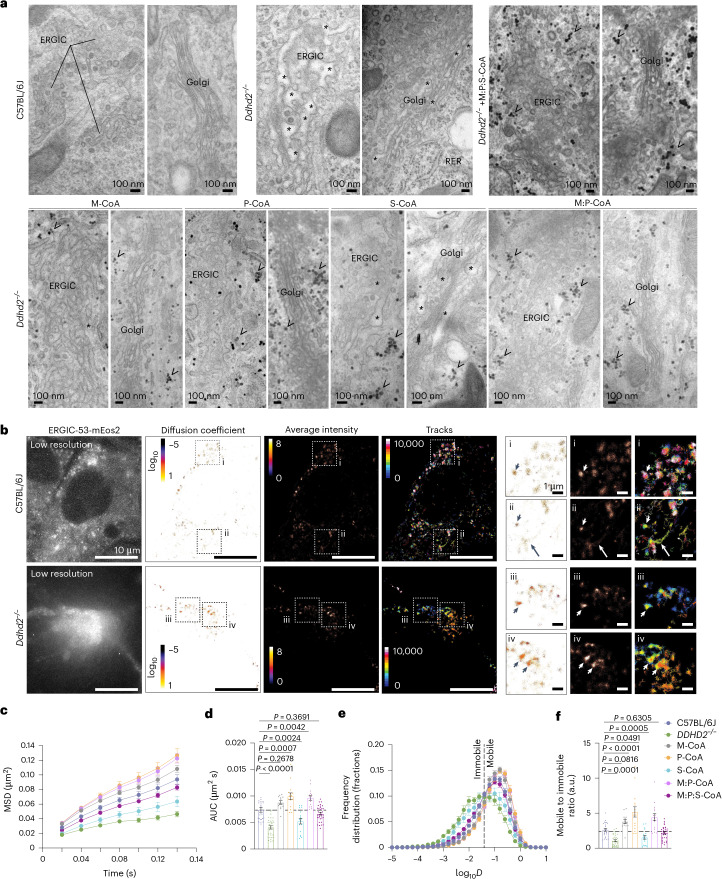
Loss of Ddhd2 alters the structure and membrane trafficking in the ERGIC and Golgi complex. **a**, Representative electron microscopy images of cultured E16 hippocampal neurons of C57BL/6J and *Ddhd2*^*−/−*^ ± 1 µM M-CoA, P-CoA, S-CoA, M:P-CoA or M:P:S-CoA for 48 h and imaged at DIV 21–22. ERGIC, Golgi complex, rough endoplasmic reticulum (RER), luminal dilation (asterisks) and glycogen (arrowheads) are indicated. See full images in Extended Data Fig. 7a. **b**, sptPALM super-resolution imaging of E16 cultured hippocampal neurons of C57BL/6J and *Ddhd2*^−/−^ transiently expressing ERGIC53-mEos2 and imaged at DIV 21–22. Low-resolution image of green fluorescence of the ERGIC53-mEos2, along with super-resolved average intensity (bar: 8 to 0, high to low density), diffusion coefficient (bar: log_10_ 1 to −5, high to low mobility) and single-molecule trajectory (bar: 0–10,000 frame acquisition) maps are show. Boxed areas (i–iv) are shown magnified on the right. Arrowheads point to confined molecules with low mobility, while arrows indicate mobile molecules. **c**–**f**, Quantification of sptPALM imaging of ERGIC53-mEos2 in E16 neuronal cultures of C57BL/6J and *Ddhd22*^−/−^ ± indicated rescues, and imaged at DIV 21–22, shown as mean square displacement (MSD, µm^2^) (**c**), area under the MSD curve (µm^2^ s) (**d**), frequency distribution of log_10_ diffusion coefficients (*D* = μm^2^ s^−1^) (**e**) and mobile-to-immobile ratio of diffusion coefficient frequency distributions (immobile log_10_*D* ≤ − 1.45 and mobile log_10_D > − 1.45) (**f**). Data are presented as mean values ± s.e.m. Dots present technical replicates. Sample sizes were *N* = 3 biologically independent experiments in each condition with 23 (C57BL/6J), 32 (*Ddhd2*^−/−^), 13 (*Ddhd2*^−/−^ + M-CoA), 15 (*Ddhd2*^−/−^ + P-CoA), 17 (*Ddhd2*^−/−^ + S-CoA), 15 (*Ddhd2*^−/−^ + M:P-CoA) and 34 (*Ddhd2*^−/−^ + M:P:S-CoA) technical replicates. The exact *P* values stated in the graphs were determined from biological replicates using ordinary one-way ANOVA Dunnett’s multiple-comparisons test (**d** and **f**). Horizontal lines in **d** and **f** indicate average C57BL/6J values.

**Extended Data Fig. 7:**
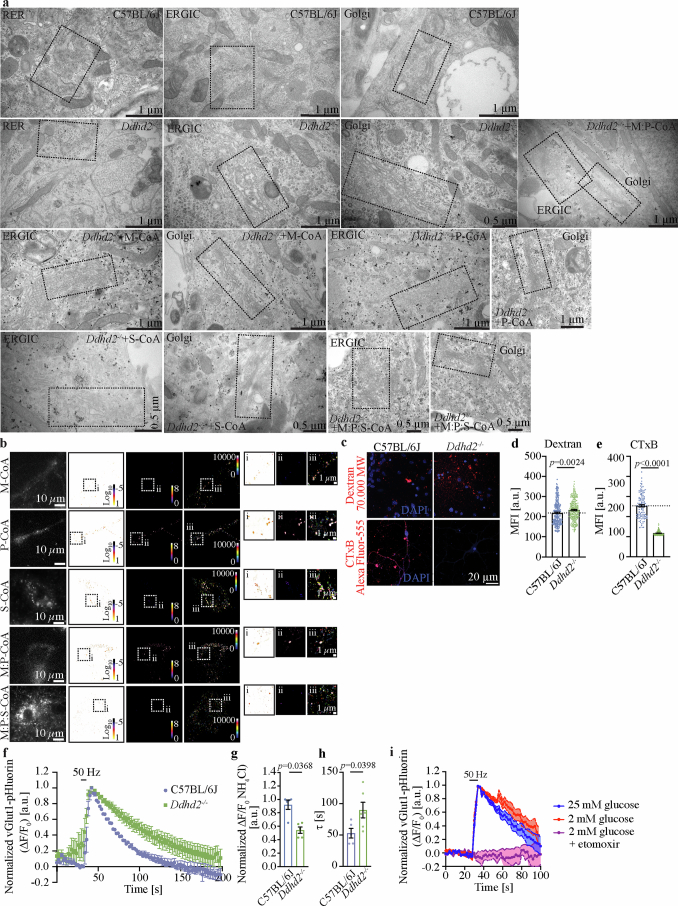
Loss of Ddhd2 leads to membrane trafficking defects in neurons. **a**, Uncropped images of the representative EM captures shown in Fig. 6a. **b**, Representative sptPALM super-resolution images of ERGIC-53-mEos2 transiently expressed in E16 *Ddhd2*^*−/−*^ hippocampal neurons treated with 1 µM fatty acyl-CoAs for 48 h as indicated and imaged at DIV21-22. Low resolution image of green fluorescence of the ERGIC-53-mEos2, along with super-resolved diffusion coefficient (bar: Log_10_ 1 to −5, high to low mobility), average intensity (bar: 8 to 0, high to low density), and single-molecule trajectory (bar: 0–10,000 frame acquisition) maps are show. Boxed areas (i-iii) are shown magnified on right. Arrowheads point to confined molecules with low mobility, while arrows indicate mobile molecules. **c**, Representative maximum projection images and quantification of mean fluorescence intensity (MFI [arbitrary units, a.u.]) of cultured E16 C57BL/6J and *Ddhd2*^*−/−*^ hippocampal neurons ( + 4 µM Ara-C) at DIV21-22 following a 5 min high K^+^ pulse in presence of **d**, 70,000 MW Dextran Tetramethylrhodamine (red) or **e**, Alexa Fluor 555-conjugated Cholera Toxin Subunit B (CTxB, red), followed by 30 min chase in low K^+^ buffer, fixation and imaging. Nuclei stained with DAPI (blue). **f**, Averaged vGlut1-pHluorin traces (ΔF/F_0_ [a.u.]), **g**, peak height of vGlut1-pH responses normalized to NH_4_Cl [a.u.], and **h**, endocytosis time-constant (τ [s]) and in C57BL/6J and responsive *Ddhd2*^*−/−*^ hippocampal neurons stimulated with a train of 300 action potentials (50 Hz, 6 s). Normalisation to the peak of each condition. **i**, Averaged vGlut1-pHluorin traces (ΔF/F_0_ [a.u.]) of C57BL/6J grown in 25 mM glucose, and 2 mM glucose ± 15 min incubation in etomoxir. Data are presented as mean values ± SEM, dots present technical replicates. N = 3 biologically independent experiments in each condition. The exact *p*-values stated in the graphs were determined from biological replicates using two-tailed Mann-Whitney test (**d, e**,) and two-tailed unpaired *t* test (**g, h**,).

Consistent with mass spectrometry findings showing altered secretory pathway protein levels in *Ddhd2*^−/−^ neurons, our previous work demonstrated that Ddhd2 loss disrupts secretory membrane trafficking in hippocampal neurons^3^. Confirming these observations, our electron microscopy analysis showed dilated luminal spaces in the endoplasmic reticulum ER–Golgi intermediate compartment (ERGIC) and Golgi complex of *Ddhd2*^−/−^ neurons, indicative of impaired membrane trafficking, while the morphology of the rough endoplasmic reticulum remained unchanged (Fig. 6a and Extended Data Fig. 7a). Except for 1 µM S-CoA, 48-h treatment with M-CoA, P-CoA, M:P-CoA and M:P:S-CoA markedly improved the dilated morphology of the ERGIC and Golgi complex in *Ddhd2*^−/−^ neurons, restoring a control-like secretory pathway organelle structure (Fig. 6a and Extended Data Fig. 7a).

To quantify membrane trafficking between the ERGIC and Golgi, single-particle tracking photo-activated localization microscopy (sptPALM)^47,48^ was used to image mannose-specific lectin ERGIC53, which functions as a cargo receptor for glycoprotein transport to and from the ERGIC, tagged with a photoactivatable mEos2 (Fig. 6b). In *Ddhd2*^−/−^ neurons, single-molecule ERGIC53-mEos2 trafficking was significantly reduced, confirming a defect in secretory membrane dynamics (Fig. 6c–f). This defect was rescued by 48-h supplementation with 1 µM M-CoA, P-CoA, M:P-CoA and M:P:S-CoA, whereas 1 µM S-CoA failed to significantly restore *Ddhd2*^−/−^ membrane trafficking (Fig. 6b–f and Extended Data Fig. 7b), indicating that bioenergetic rescue was not sufficient in restoring these defects and further suggesting additional roles for these sFFAs beyond energy fuelling.

### Loss of Ddhd2 perturbs exocytosis and endocytosis kinetics and alters plasma membrane fluidity

**Fig. 7:**
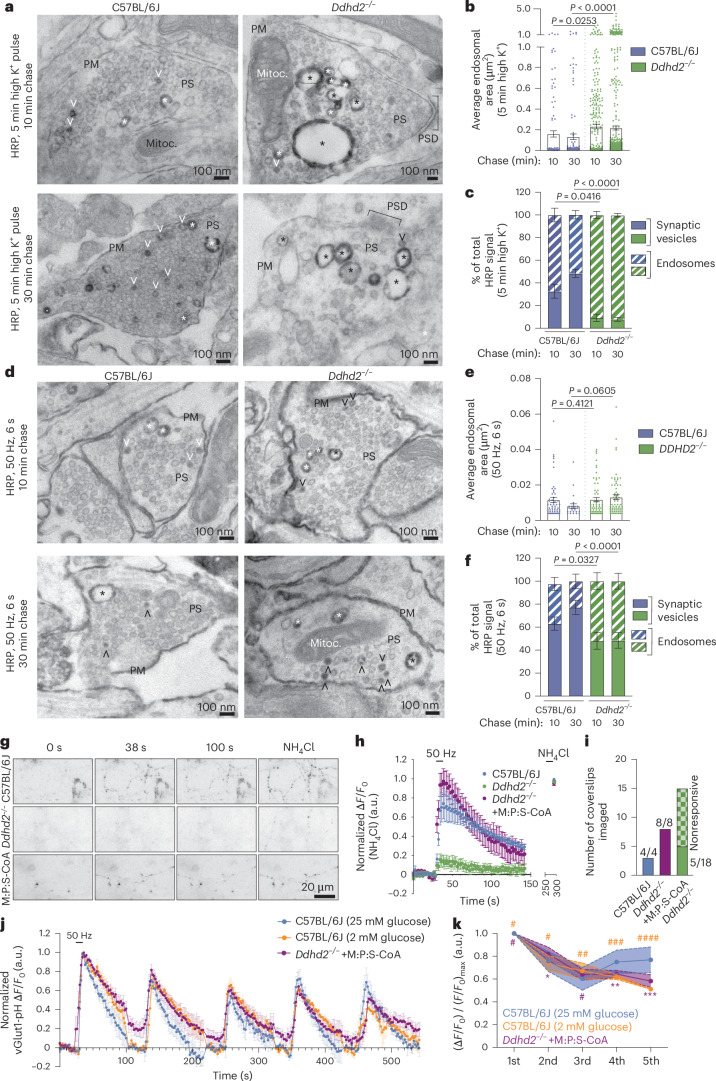
Ddhd2 is required for efficient synaptic vesicle exocytosis and endocytosis. **a**, Representative electron microscopy images of E16 C57BL/6J and *Ddhd2*^−/−^ cultured hippocampal neurons pulsed with 5 min high K^+^ in the presence of HRP and chased for either 10 min or 30 min, followed by cytochemical staining (that is, dark precipitate) and preparation for electron microscopy at DIV 21–22. Large endosomes (asterisks), synaptic vesicles (open arrowheads), plasma membrane (PM), mitochondria (Mitoc.) and PSD in the presynapses (PS) are indicated. **b**,**c**, Quantification of average sectional area (µm^2^) of large endosomes (**b**) and HRP-stained synaptic vesicles (SVs) and endosomes (**c**) shown as a percentage of the total in indicated conditions quantified from electron microscopy images. **d**, Representative electron microscopy images of cultured C57BL/6J and *Ddhd2*^−/−^ neurons challenged with 300 action potentials (50 Hz, 6 s) in the presence of HRP, followed by either 10 min or 30 min chase, followed by cytochemical staining (that is, dark precipitate) and preparation for electron microscopy at DIV 21–22. **e**,**f**, Quantification of average sectional area (µm^2^) of large endosomes (**e**) and HRP-stained synaptic vesicles (SVs) and endosomes (**f**) shown as a percentage of the total in indicated conditions quantified from electron microscopy images. **g**, Representative widefield time-series images of E16 cultured hippocampal C57BL/6J and *Ddhd2*^−/−^ (±1 µM M:P:S-CoA for 48 h) neurons transiently expressing vGlut1-pHluorin at rest (baseline), following 300 action potentials (50 Hz, 6 s) and NH_4_Cl dequenching. **h**, vGlut1-pHluorin traces (Δ*F/F*_0_) of C57BL/6J and *Ddhd2*^−/−^ ± 1 µM M:P:S-CoA for 48 h, normalized to NH_4_Cl response peak in each condition. **i**, Number of indicated neuronal cultures responding to stimulation with 300 action potentials (50 Hz, 6 s). **j**, vGlut1-pHluorin traces (Δ*F/F*_0_) of C57BL/6J and *DDHD2*^−/−^ + 1 µM M:P:S-CoA for 48 h following five repetitive stimulations at 50 Hz, at 2-min intervals, normalized to the first stimulation peak in each condition allowing the assessment of endocytosis kinetics. **k**, Summary data of normalized vGlut1-pHluorin peak responses over five repetitive stimulations. Data are presented as mean values ± s.e.m. Dots in **b** and **c** show the endosomal area obtained from technical replicates. Sample sizes were *N* = 3 (**b**, **c**, **e**, **f**, **h** and **i**), *N* = 5 (**j**, **k**; C57BL/6J 25 mM glucose), *N* = 4 (**j**, **k**, C57BL/6J 2 mM glucose) and *N* = 6 (**j**, **k**; *Ddhd2*^−/−^ + M:P:S-CoA) biologically independent experiments in each condition. The exact *P* values stated in the graphs were determined from biological replicates using one-way ANOVA Kruskal–Wallis multiple-comparison test (**b** and **e**), with one-way ANOVA Sidak’s multiple-comparisons test (**c** and **f**), two-way ANOVA Sidak’s multiple-comparisons test (**k**; where ^#^*P* > 0.9999, ^##^*P* = 0.9986, ^###^*P* = 0.8518, ^####^*P* = 0.0929, *P = 0.9976, **P = 0.9277 and ***P = 0.3088 compared to C57BL/6J neurons cultured in 25 mM glucose).

Exocytosis at synapses is tightly coupled to endocytic membrane retrieval, ensuring neurotransmission fidelity and synaptic integrity^49,50^. Given the lowered synaptic ATP levels, impaired response to high K⁺ stimulation (Fig. 4e,f) and altered synaptic proteome (Fig. 5g) in *Ddhd2*^−/−^ neurons, synaptic vesicle recycling was investigated using quantitative electron microscopy. Hippocampal neurons were pulsed with horseradish peroxidase (HRP) for 5 min in high K⁺ and chased in low K⁺ for 10 min or 30 min before cytochemical staining (produces a dark electron-dense HRP precipitate) and electron microscopy visualization (Fig. 7a). In *Ddhd2*^−/−^ neurons, HRP-positive endosomes were significantly larger, and HRP-labelled synaptic vesicles were rarely observed compared to controls (Fig. 7a–c), indicating impaired vesicle recycling. Electric field stimulation (300 action potentials, 50 Hz) further confirmed HRP accumulation in enlarged endosomes in *Ddhd2*^−/−^ neurons as opposed to synaptic vesicle uptake of HRP, which was observed in control neurons (Fig. 7d,e). Furthermore, control neurons showed that the HRP internalized in larger endosomes was gradually entering synaptic vesicles over time, a process that was impaired in *Ddhd2*^*−/−*^ neurons (Fig. 7f). Pulse-chase uptake assays with 70-kDa dextran and cholera toxin subunit B (CTxB) further supported this, showing significantly increased bulk endocytosis and reduced clathrin-mediated uptake in *Ddhd2*^−/−^ neurons (Extended Data Fig. 7c–e). These findings indicate disrupted balance between synaptic vesicle recycling and bulk endocytosis in *Ddhd2*^−/−^ neurons, suggesting compromised fidelity of neurotransmission.

**Extended Data Fig. 8:**
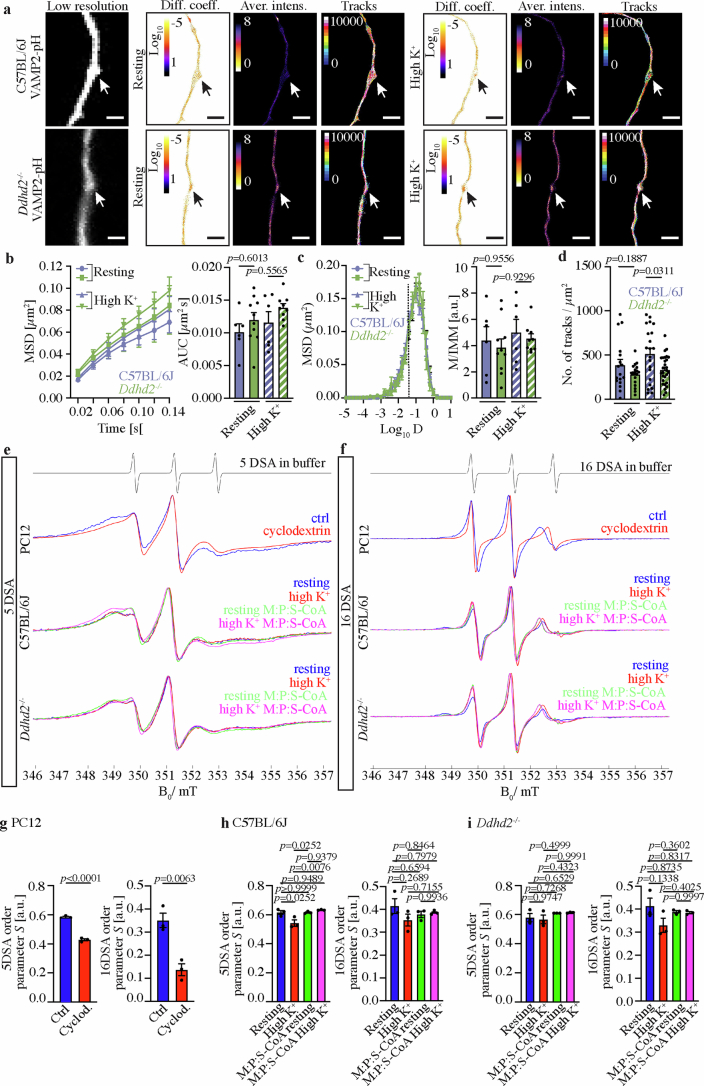
Protein trafficking and membrane fluidity. **a**, uPAINT super-resolution imaging VAMP2-pHluorin (VAMP2-pH) bound Atto647N-nanobodies in cultured C57BL/6J and *Ddhd2*^*−/−*^ neurons in resting condition (low K^+^) and following high K^+^ stimulation. Low resolution images of green fluorescence of the VAMP2-pHluorin, along with super-resolved diffusion coefficient (bar: Log_10_ 1 to −5, high to low mobility), average intensity (bar: 8 to 0, high to low density), and single-molecule trajectory (bar: 0–10,000 frame acquisition) maps are show. Arrows point to presynapses. Quantification of single-molecule mobility of VAMP2-pH/Atto647N-nanobodies in C57BL/6J and *Ddhd2*^*−/−*^ neurons shown as **b**, mean square displacement (MSD [µm^2^]) and area under the MSD curve (µm^2^ s), **c**, frequency distribution of log_10_ diffusion coefficients ([D] = μm^2^ s^−1^), and mobile to immobile ratio of diffusion coefficient frequency distributions (immobile Log_10_D ≤ − 1.45 and mobile Log_10_D > − 1.45 [a.u.]) in indicated conditions. **d**, Quantification of single-molecule numbers of VAMP2-pH/Atto647N-nanobodies per area (µm^2^) in C57BL/6J and *Ddhd2*^*−/−*^ neuron presynapses in resting and high K^+^ stimulated conditions. **e, f**, X-band (9.852) CW EPR_spectra_ recorded from neuronal cultures of E16 C57BL/6J and *Ddhd2*^*−/−*^ with and without 1 µM M:P:S-CoA-treatment for 48 h and analysed at DIV21-22 in resting condition (non-stimulated) and following a 5-min high K^+^ stimulation using a microwave power of 16 mW and a modulation amplitude of **e**, 0.3 mT (5DSA) and **f**, 0.1 mT (16DSA). PC12 neurosecretory cells treated with 1 µM cyclodextrin (that is cholesterol scavenger) or DMSO (vehicle media; Ctrl) for 30 min are shown as a positive control for a decrease in membrane order, and therefore an increase in membrane fluidity. Quantification of 0.3 mT (5DSA) and 0.1 mT (16DSA) membrane order parameter *S* [a.u.] in **g**, PC12 cells, **h**, C57BL/6J and **i**, *D*^*−/−*^ neurons in indicated conditions. Data are presented as mean values ± SEM, dots present technical replicates in **b-d**, and biological replicates in **g-i**,. N = 3 biologically independent experiments in each condition. The exact *p*-values stated in the graphs were determined from biological replicates using ordinary one-way ANOVA Šídák’s multiple comparisons test (**b, c, d**, **h, i**,) and two-tailed unpaired *t* test (**g**,).

To monitor synaptic neurotransmission and vesicle recycling, we used vesicular glutamate transporter 1 pHluorin (vGlut1-pH)^51,52^, which is a pH-sensitive reporter that is quenched in the acidic synaptic vesicle lumen and becomes fluorescent following exocytosis and exposure to neutral pH. High-frequency stimulation (50 Hz, 6 s) revealed that most *Ddhd*^−/−^ neurons failed to respond to stimulation (Fig. 7g–i). Remarkably, 48-h M:P:S-CoA treatment restored responsiveness of *Ddhd*^−/−^ neurons to control levels (Fig. 7h,i). Responding *Ddhd2*^−/−^ neurons showed lower vGlut1-pH peak responses and slower retrieval into acidic vesicles indicated by increased time constant value τ (Extended Data Fig. 7f–h). To investigate the extend of which nerve terminals could sustain synaptic vesicle recycling, multiple rounds of stimulation were introduced. Five repetitive 300 action potentials (50 Hz, 6 s) in C57BL/6J neurons (grown in 2 mM, or standard 25 mM, glucose culture media) slowed synaptic vesicle endocytosis and showed signs of exocytosis run down by the fifth stimulation (Fig. 7j). M:P:S-CoA-treated *Ddhd2*^−/−^ neurons maintained vesicle recycling similarly to low-glucose C57BL/6J neurons, while high-glucose C57BL/6J neurons showed slightly faster kinetics (Fig. 7j). Peak amplitudes recorded from high-glucose C57BL/6J neurons were not significantly different from the other conditions (Fig. 7k). Supporting the role of β-oxidation in synaptic function, acute etomoxir treatment abolished responses in C57BL/6J neurons (Extended Data Fig. 7i), mimicking the *Ddhd2*^−/−^ phenotype (Fig. 7h). Impaired trafficking in *Ddhd2*^−/−^ neurons was also observed using universal point accumulation in nanoscale topography live-cell super-resolution imaging^53^ of pHluorin-tagged vesicle-associated membrane protein 2 (VAMP2-pH) targeted with anti-GFP Atto647N nanobodies on the neuronal plasma membrane in resting conditions and following stimulation. Although the nanoscale mobility of VAMP2-pH/Atto647N was similar in control and *Ddhd2*^−/−^ neurons in both conditions (Extended Data Fig. 8a–c), the number of VAMP2 molecules on the plasma membrane was significantly reduced in *Ddhd2*^−/−^ neurons (Extended Data Fig. 8d), suggesting an imbalance in VAMP2 synaptic vesicle recycling. Together, these findings underscore the importance of β-oxidation in sustaining neurotransmission and the rescue potential of fatty acyl-CoA supplementation.

Ddhd2 activity-dependent production of sFFAs may alter membrane lipid composition, affecting fluidity and synaptic vesicle recycling. The FFA balance, which contributes to membrane fluidity^39^, is tightly controlled in neurons to maintain normal cellular functions, including intracellular trafficking and exocytosis^38^. We investigated if the observed sFFA imbalance in *Ddhd2*^−/−^ neurons^3^ disturbed membrane order using electron paramagnetic resonance (EPR). The membrane order parameter (S) was calculated from EPR spectra using nitroxyl radical probes 5-DSA and 16-DSA, which measure fluidity dynamics (fluidity) of the membrane near the protein–aqueous interface and hydrophobic core, respectively^54^. As a control, cyclodextrin-treated PC12 cells showed decreased S due to cholesterol extraction^55^ (Extended Data Fig. 8e–g). In C57BL/6J neurons, high K⁺ stimulation reduced membrane order at the surface (5-DSA), an effect absent in *Ddhd2*^−/−^ neurons (Extended Data Fig. 8e,f,h,i), suggesting a Ddhd2-dependent modulation of the plasma membrane. Fatty acyl-CoA supplementation had no effect at rest but prevented stimulation-induced changes in control neurons (Extended Data Fig. 8h,i), indicating potential feedback inhibition of Ddhd2 by sFFAs or their metabolites. No significant changes were observed with 16-DSA. These findings suggest Ddhd2 regulates plasma membrane fluidity during neuronal activity and may be modulated by its own products, as shown for other lipases^56^.

### Combined supplementation of M:P:S-CoA restores *Ddhd2*^−/−^ synaptic structure and vesicle recycling defects

**Fig. 8:**
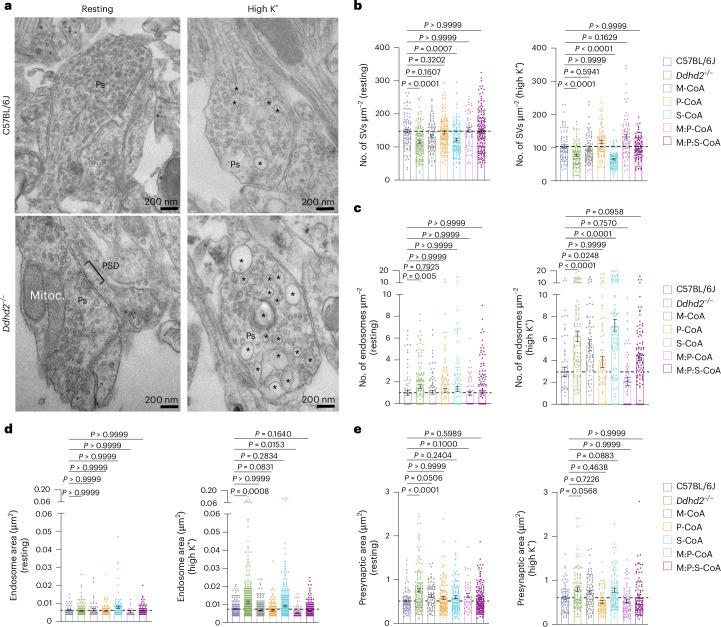
Perturbed presynaptic membrane trafficking in cultured *Ddhd2*^−/−^ hippocampal neurons is restored with M:P:S-CoA supplementation. **a**, Representative electron microscopy images. **b**–**e**, Quantification of number of synaptic vesicles (SVs) (**b**) and endosomes (**c**) per presynaptic area (µm^2^), endosomal area (µm^2^) (**d**) and presynaptic area (µm^2^) (**e**) in cultured E16 hippocampal C57BL/6J and *Ddhd2*^−/−^ ± 1 µM M-CoA, P-CoA, S-CoA or combinations for 48 h, fixed, processed for electron microscopy, and imaged at DIV 21–22. Representative images of presynapses (Ps), endosomes (asterisks), mitochondria (Mitoc.) and PSD in the resting (non-stimulated) condition and following a 5-min high K^+^ stimulation are indicated. Data are presented as mean values ± s.e.m. Dots in **b** and **c** show the number of synaptic vesicles and endosomes, respectively, per synapse, and in **d** and **e**, endosomal and presynaptic area, obtained from technical replicates. Sample size is *N* = 3 biologically independent experiments in each condition. The exact *P* values stated in the graphs were determined from biological replicates using a one-way ANOVA Kruskal–Wallis multiple-comparison test (**b**–**e**). Horizontal lines in **b**–**e** indicate average C57BL/6J values.

To investigate whether fatty acyl-CoA supplementation could rescue synaptic defects, we used quantitative electron microscopy to assess presynaptic vesicle and endosome numbers, and the size of endosomes and presynapses, in resting and stimulated neurons (Fig. 8a). M:P:S-CoA treatment restored synaptic vesicle numbers in *Ddhd2*^−/−^ neurons to control levels under both conditions (Fig. 8b). M-CoA and P-CoA or combinations of the two, but not S-CoA, also improved vesicle numbers at rest and after stimulation in *Ddhd2*^−^^/^^−^ neurons to levels comparable to control (Fig. 8b). All treatments except S-CoA reduced elevated endosome numbers (Fig. 8c) and endosome size after stimulation (Fig. 8d), restoring the presynaptic area to control levels (Fig. 8e) in *Ddhd2*^−/−^ neurons, underscoring the reversibility of these defects by fatty acyl-CoA treatments. Taken together, these findings demonstrate that the activity-dependent release of sFFAs by Ddhd2 plays a crucial role in synaptic vesicle recycling and maintaining presynaptic integrity. These results also indicated that the administration of fatty acyl-CoAs, in particular M-CoA, P-CoA or a combination of the two, can facilitate restoring the balance between activity-dependent bulk endosomes and synaptic vesicle recycling and the synaptic functionality.

## Discussion

### Discovery of a Ddhd2-dependent metabolic pathway that fuels neuronal β-oxidation and energy

Current models of neuronal activity, memory consolidation, and neurological disease are largely proteocentric, while the role of lipid metabolism in brain function remains underexplored^4,57,58^. We recently identified Ddhd2 as a key neuronal regulator that releases sFFAs, mainly myristic, palmitic and stearic acids, in response to stimulation in vitro, and in vivo following energy-demanding learning tasks in wild-type neurons^3^. Loss of the Ddhd2-dependent sFFA fluxes in mice leads to progressive memory and motor deficits^3^ resembling HSP54. The physiological role of the activity-dependent sFFA release has remained unclear. Here, we show that Ddhd2 releases sFFAs to fuel mitochondrial β-oxidation and ATP production, challenging the long-held belief that under normal conditions neurons rely solely on glucose or glucose metabolites for energy^16^. This pathway operates both at rest, supporting ~20% of neuronal energy needs, and during activity, when sFFA flux increases^3^ to sustain neurotransmission. Ddhd2 deficiency impairs this energy homeostasis. Based on the synaptic ATP sensor experiments, following high K^+^ stimulation and inhibition of glycolysis with 2-DG, the remaining ATP levels, which are supported by mitochondrial β-oxidation, were 40% of the total in C57BL/6J and 33% in *Ddhd2*^−/−^ neurons. These results indicate that some level of β-oxidation remains in *Ddhd2*^−/−^ neurons, which is supported by our previous FFA lipidomics, showing smaller but detectable levels of sFFAs at basal levels in *Ddhd2*^−/−^ mice^3^.

Our findings indicate an inverse relationship between glycolysis and β-oxidation: when β-oxidation is active, glycolysis decreases and glycogen accumulates at high glucose concentrations, whereas its inhibition elevates glycolysis. Elevated levels of fatty acyl-CoA or metabolites that indicate a high energy state may inhibit DDHD2 through product feedback, limiting excessive lipid breakdown, as observed with other lipases^56^. Given the brain’s metabolic vulnerability and changes in the FFA composition during ageing^59,60^, and the metabolic changes in conditions like Alzheimer’s disease^61^, our discovery of neurons fuelling β-oxidation with endogenously released sFFAs, which can be carefully supplemented externally in times of low fatty acyl abundance, could broadly impact human health and guide future work in this area. This work represents a paradigm shift in understanding brain energy metabolism and underscores the essential role of sFFA metabolism in cognitive function.

### Revisiting neuronal metabolism

The long-standing view that neurons rely exclusively on glucose for energy stems largely from studies using isolated mitochondrial preparations, which assessed FFA oxidation under conditions that did not account for neuronal activity or endogenous sFFA production by Ddhd2. These studies concluded that neuronal β-oxidation is low, typically contrasting neurons against cells with robust β-oxidation such as astrocytes^62^ that also express higher levels of Cpt1a mRNA^63^ than neurons. For example, rat brain mitochondria show significantly lower activity levels of β-oxidation enzymes, 0.7% thiolase, 50% acyl-CoA dehydrogenase and 19% enoyl-CoA hydratase, compared to heart mitochondria^64^. Moreover, radioactive labelling studies suggest that neurons do not oxidize short-chain FFAs^65^. However, these findings do not exclude neuronal oxidation of endogenous long-chain FFAs or its role in meeting high energy demands. Supporting this, we recently showed that neurons, not astrocytes, mediate Ddhd2-dependent sFFA-driven responses crucial for memory consolidation^3^. Further evidence includes oxidation of palmitoyl-carnitine by isolated neuronal mitochondria^66^ and increased OCR following FFA supplementation in human neurons^67^. In addition, inhibition of long-chain fatty acid mitochondrial import with etomoxir leads to a remarkably similar reduction in the rates of mitochondrial OCR in astrocytes (approximately 35%) and neurons (20%)^63^.

### Disease relevance and potential therapeutic impact

In humans, biallelic mutations of *DDHD2* cause HSP54, a disorder characterized by progressive memory impairment and motor neuron dysfunction^6,13,15,39,41^. The cellular mechanisms linking DDHD2 loss to HSP54 progression have remained poorly understood, and there are currently no effective treatments or cures. Our research reveals a complex neuropathology resulting from the loss of DDHD2 function demonstrating impacts that extend beyond the immediate enzymatic targets, showing deficiencies in neuronal energy metabolism and mitochondrial integrity, proteostasis, secretory pathway membrane trafficking and synaptic function. While the energy losses in *Ddhd2*^−/−^ neurons, and in DDHD2^W103R^-expressing *Ddhd2*^−/−^ neurons mimicking HSP54, were efficiently rescued with all tested fatty acyl-CoAs, the balance in proteostasis, mitochondrial structure, secretory pathway membrane trafficking and synaptic function was only partially restored with the bioenergetic rescue, while the combined M:P:S-CoA treatment efficiently restored all these defects to the levels of control neurons. These results indicate that coordinated release and ratios of myristic, palmitic and stearic acids (released in neurons in a ratio of 1:0.76:0.88 following neuronal activity^3^, respectively) are tightly controlled in neurons. Therefore, the balance between myristic and palmitic acids (perhaps through N-myristoylation and S-palmitoylation of numerous neuronal proteins^20,68,69^) and stearic acid (shown to have a role in shielding neurons from oxidative stress^70^) plays an important role in the neurons. Our results also indicate that Ddhd2 may play a role in regulating membrane fluidity during neuronal activity, and that its function may be modulated by its own metabolic products, as shown for other lipases^56^. Whether these findings are a direct effect of Ddhd2 activity, or a downstream effect of the altered proteostasis or other factors, remains to be studied in the future. Together, these results suggest that complex neuropathology resulting from the loss of Ddhd2 is partially due to energy loss, and partially due to downstream non-energetic roles of the sFFAs. Moreover, our results show that preconjugation of fatty acids with CoA restores the neuronal energy balance more efficiently than fatty acids without CoA activation, without causing oxidative stress. How these activated fatty acids are internalized in neurons and what is the mechanism of enhanced rescue compared to fatty acids without CoA conjugation remains to be studied in the future. Preconjugated fatty acyl-CoAs are water soluble, as opposed to FFAs that can only be solubilized in dimethylsulfoxide or methanol, making M:P:S-CoA treatment an attractive choice for future therapeutic testing. Together, our findings identify a potential therapeutic avenue for HSP54 and, potentially, other neurological conditions marked by FFA imbalances.

### Limitations of the study

The current study presents an in vitro investigation of the Ddhd2-mediated metabolic pathway in rodent primary cultured neurons. Although Ddhd2 is a highly conserved phospholipase in mammals, additional studies are needed to confirm the sFFA-powered metabolic pathway in human neurons and to reveal the therapeutic potential of fatty acyl-CoA in vivo.

## Methods

### Animals and ethics

All animal procedures complied with the Australian Code of Practice for the Care and Use of Animals for Scientific Purposes and were approved by the University of Queensland Animal Ethics Committee (AE000770, AE000209). *Ddhd2* knockout (*Ddhd2*^−/−^) mice^1^ on the C57BL/6J background were obtained from the Scripps Research Institute (USA). Wild-type C57BL/6J mice were maintained in-house at the Queensland Brain Institute (from Jackson Laboratory, strain no. 000664, RRID: IMSR_JAX:000664). Mice were housed in a PC2 facility under a 12-h light–dark cycle (80% intensity), 18–24 °C, 30–70% humidity with ad libitum access to food and water. Wild-type NMRI mice were obtained from Charles River and housed at the University of Helsinki under European legislation for animal use in research (license nos. KEK21-012 and KEK24-013, University of Helsinki).

### Antibodies

For immunofluorescence, antibodies used were: GFAP (Abcam, ab7260, RRID: AB_305808), MAP-2 (Synaptic Systems, 188004, RRID: AB_2138181), synapsin 1 (Synaptic Systems, 106011, RRID: AB_2619772), TOMM20 (Abcam, ab186734, RRID: AB_2716623), Alexa Fluor 647 anti-rabbit IgG (Thermo Fisher Scientific, A-21245, RRID: AB_2535813), Alexa Fluor 488 anti-mouse IgG (Thermo Fisher Scientific, A32723, RRID: AB_2633275) and anti-Rabbit IgG Alexa Fluor Plus 647 (Thermo Fisher Scientific, A32733, RRID: AB_2633282). For western blotting, antibodies used were: GFAP and MAP-2 (as above), DDHD2 (Proteintech, 25203-1-AP, RRID: AB_2879957), β-actin (Sigma-Aldrich, A5316; RRID: AB_476743), IRDye 680RD Goat anti-Mouse IgG Secondary Antibody (Licorbio, 926-68070, RRID: AB_10956588), IRDye 800CW Goat anti-Rabbit IgG Secondary Antibody (Licorbio, 926-32211, RRID: AB_621843) and IRDye 800CW Donkey anti-Guinea Pig IgG Secondary Antibody (Licorbio, 926-32411, RRID: AB_1850024).

### Primary neuron and neuron–astroglia cultures, transfections and treatments

Pregnant wild-type C57BL/6J (The University of Queensland in-house mouse strain) and *Ddhd2*^−/−^ mice were killed via CO_2_ and cervical dislocation. Hysterectomy was done to collect E16 embryos. Isolated brain dissection from cortices and hippocampi from embryos were combined (that is, mixed cultures, sex unknown), prepared, cultured and transfected as previously described in a stepwise protocol^71^. In short, dissected cortices or hippocampi from E16 embryos were collected in HBSS (Gibco, 14185-052), digested with 2.5% trypsin (Gibco, 15090-046) at 37 °C for 10 min, and then treated with 10% fetal bovine serum (FBS; Gibco, 26140-079) and 1% deoxyribonuclease I (Sigma-Aldrich, D5025-375KU) for 10 min at 37 °C. Tissue was homogenized and centrifuged at 120*g* for 7 min at room temperature. The pellet was then resuspended in Neurobasal medium (Gibco, 21103049) supplemented with 1× GlutaMAX (Gibco, 35050-061), 10% FBS and penicillin–streptomycin (100 U ml^−1^ and 100 μg ml^−1^, respectively; Gibco, 15140-122). Neurons were seeded at 100,000 cells per 78.54 mm² on poly-l-lysine (PLL) hydrobromide-coated (Sigma-Aldrich, P2636; 0.1 mg ml^−1^) dishes (CellVis, D29-20-1.5-N, 1 mg ml^−1^), or at equivalent density on other plate formats. After 4–6 h, medium was replaced with serum-free Neurobasal medium containing 1× GlutaMAX, penicillin–streptomycin and 1× B27 (Gibco, 17504-044) and thereafter one-third of the medium was renewed every 3 days. To generate neuronal cultures, 4 µM Ara-C (Sigma, C1768) was added from DIV 3 onwards. Cultures without Ara-C treatment contain neuron–astroglia cultures. The cultures were maintained at 37 °C in a humidified incubator with 95% air and 5% CO_2_ until DIV 21–22, unless otherwise stated, when experiment were carried out. Neurons were transfected at DIV 14 using Lipofectamine 2000 Transfection Reagent (Thermo Fisher Scientific, 11668019) according to the manufacturer’s instructions. Cultured C57BL/6J and *Ddhd2*^−/−^ neurons were treated for 48 h on DIV 18–20 with 1 µM myristic acid (70082), myristoyl-CoA (M-CoA; M4414), palmitoyl-CoA (P-CoA; P9716), stearoyl-CoA (S-CoA; S0802), M:P-CoA (1:1 ratio of M-CoA and P-CoA) or M:P:S-CoA (1:0.76:0.88 ratio of M-CoA, P-CoA and S-CoA as previously described^3^; all from Sigma-Aldrich).

Pregnant wild-type NMRI (Charles River) mice were killed via CO_2_ and cervical dislocation (KEK24-013, University of Helsinki). Hysterectomy was done to collect E15.5–16.5 embryos. Cortices from the embryos were dissected as previously described^72^. Dissected cortices were pooled, rinsed three times with Ca^2+^/Mg^2+^-free HBSS (Gibco, 14175-053), incubated on ice (10 min) and digested with 2.5% trypsin (MP, 103139) for 15 min at 37 °C. Digestion was neutralized with HBSS containing 10% FBS (Gibco, 10500056) and DNase I (Roche, 11284932001), followed by brief centrifugation (30 s at 38*g*) and two washes in 10% FBS HBSS (centrifugation between washes for 30 s at 38*g*). Cells were dissociated in Neurobasal medium (Gibco, 21103049) by trituration with a P1000 pipette, centrifuged at 98*g* (1 min), resuspended, triturated again and spun at 24*g* for 30 s. The supernatant was collected, centrifuged at 98*g* for 2 min and resuspended in fresh medium. Cells were cultured in Neurobasal medium supplemented with 20% B27 (Gibco, 17504-044), 0.25% l-glutamine (Gibco, 25030-024) and 0.2% Primocin (Invitrogen, ant-pm). Cells were seeded on 0.01% PLL-coated plates (Bio-Techne Cultrex, 3438-100-01) at 1–2 × 10^6^ cells per well (of a six-well plate) or 14.6 × 10^6^ cells per plate (10 cm) densities. Cultures were maintained at 37 °C and 5% CO_2_ with half of the medium changed every 3–4 days until sample collection.

### Cell cultures

PC12 cells (American Type Culture Collection, CRL-1721), HEK293T cells (American Type Culture Collection, CRL-3216) and Gibco Viral Production Cells (Gibco, A35347) were cultured according to the manufacturer’s instructions. All cell lines were confirmed to be negative for mycoplasma in prior experiments.

### Construction of FUW-DDHD2^W103R^-myc

pFLAG–DDHD2 wild-type, W103R, D660H and S351A^73^ were a kind gift from Y. Maemoto (Tokyo University of Pharmacy and Life Sciences, Japan). For the cloning of FUW-DDHD2^WT^-myc and FUW-DDHD2^D660H^-myc and FUW-DDHD2^S351A^-myc, the PCR fragment was digested with XbaI (NEB, R0145S) and AscI (NEB, R0558L). The primers are listed in Supplementary Table 6.

Plasmid encoding the *DDHD2* Trp103Arg (W103R) mutation was generated using the overlapping PCR protocol with the primers listed in Supplementary Table 6. The overlap fragment was inserted into the pFLAG–DDHD2 plasmid through the MluI (NEB, R3198S) and BstbI (NEB, R0519L) restriction sites. cDNAs encoding DHHD2 were amplified using the primers listed in Supplementary Table 6.

### Construction of pmEOS2-C1-ERGIC53

The pmEOS2-C1-ERGIC53 plasmid was constructed by amplifying a PCR fragment of ERGIC53 of the pMXs-IP spGFP-ERGIC53 using restriction enzymes BamHI (NEB, R0136S) and NheI (NEB, R3131S) on the pMXs-IP spGFP-ERGIC53 plasmid. pMXs-IP spGFP-ERGIC53 was a gift from N. Mizushima^74^. The primers used are listed in Supplementary Table 6. The PCR fragment was digested with BamHI and NheI restriction endonucleases, and then ligated to the pmEos2-C1 plasmid backbone digested with XhoI and BamHI restriction endonucleases. The pmEOS2-C1-ERGIC53 plasmid was constructed using the In-Fusion Snap Assembly Cloning Kit (Takara, 638943) and transformed into OmniMAX competent cells (Invitrogen, C854003). Overnight cultures of the positive clones were grown in LB Kanamycin (30 μg ml^−1^) media and plasmid DNA was extracted using the QIAprep Spin Miniprep Kit (Qiagen, 27104). Purified plasmid DNA was sequenced using ABI BigDye Terminator v3.1 Sequencing (Thermo Fisher Scientific) at the Genome Research Facility (AGRF) with the primers listed in Supplementary Table 6. Data analysis was performed using the software SnapGene 5.3 (https://www.snapgene.com/updates/snapgene-5-3-3/).

### Lentiviral production

Lentiviral particles were generated by transfecting HEK293T cells by the calcium–phosphate co-precipitation method with 7 mg of the plasmid of interest, 3 mg each of pMD2.G envelope plasmid (a gift from D. Trono; RRID: Addgene_12259)^75^, pRSV-Rev encoding plasmid (a gift from D. Trono, Addgene plasmid, 12253)^75^ and pMDLg/pRRE (a gift from D. Trono, Addgene plasmid, 12251 packaging construct)^75^. Forty-eight hours after transfection, lentivirus-containing supernatant was collected, and lentiviral particles were harvested using the polyethene glycol precipitation solution, followed by centrifugation at 1,500*g* for 30 min. Concentrated viruses were resuspended in Neurobasal medium, flash frozen in liquid nitrogen and stored at −80 °C.

### AAV9 production for synaptic ATP sensors

pAAV.hSynapsin.(cyto).cpSFGFP.HaloTag was a gift from T. Brown and the HHMI-JRC Tool Translation Team (Addgene plasmid, 209666)^34^. pAAV.hSynapsin.(cyto).iATPSnFR2.A95K.HaloTag was a gift from T. Brown and the HHMI-JRC Tool Translation Team (Addgene plasmid, 209664)^34^. Recombinant AAV vector was produced by The University of Queensland’s Viral Vector Core facility as previously described^76^, with modifications to incorporate the AAV-MAX production system (Thermo Fisher Scientific), according to the manufacturer’s instructions. In brief, Gibco viral production cells were diluted to a density of 3 × 10^6^ cells per ml and transfected with three constructs: (1) pAAV.hSynapsin.(cyto).cpSFGFP.HaloTag or pAAV.hSynapsin.(cyto).iATPSnFR2.A95K.HaloTag, (2) pHelper and (3) the rep2/cap9 plasmid along with the RevIT AAV enhancer (Mirus Bio). Viral particles were harvested and purified using PEG precipitation, chloroform extraction, two-phase separation in aqueous solution and discontinuous gradients of iodixanol. The AAV was then concentrated using a Vivaspin 100-kDa centrifugal filter tube. AAV was quantified using the Bio-Rad QX200 droplet digital PCR system (Expert Design Assay: AAV, ITR-2, assay ID dEXD15274642).

### ATP and acetyl-CoA detection

ATP levels were measured using the Luminescent ATP Detection Assay Kit (Abcam, ab113849) following the manufacturer’s protocol. For resting and stimulated conditions, neurons were incubated in low K⁺ buffer consisting of 0.5 mM MgCl_2_ (Chem-Supply, MA029), 2.2 mM CaCl_2_ (Sigma-Aldrich, C5080), 5.6 mM KCl (Ajax Finechem, 1206119), 145 mM NaCl (Amresco, X190), 5.6 mM d-glucose (Amresco, 0188), 0.5 mM ascorbic acid (Sigma-Aldrich, A5960), 0.1% BSA (Sigma-Aldrich, A7638), 15 mM HEPES (Gibco, 15630-080) at pH 7.4 and 290–310 mOsm, or high K⁺ buffer (same as low K^+^ except for 56 mM KCl, 95 mM NaCl) for 5 min at 37 °C, in a 5% CO_2_ cell culture incubator, after which the ATP levels were quantified according to manufacturer’s protocol. For quantification of the effect of M-CoA and M-CoA + etomoxir (MedChemExpress, HY-50202), ATP levels in each of the biological replicates were normalized to *Ddhd2*^−/−^. Acetyl-CoA levels were measured using the Acetyl-Coenzyme A Kit (Sigma-Aldrich, MAK039-1KT) and quantified against a standard curve per the manufacturer’s instructions. Brain tissue (20 mg) from adult female C57BL/6J and *Ddhd2*^−/−^ mice was flash frozen in liquid nitrogen and pulverized for the analysis.

### Immunofluorescence staining, imaging and analysis

For analysis of glial content, E16 hippocampal neurons (Ara-C) were cultured on 96-well glass-bottom dishes (Cellvis), fixed with 4% paraformaldehyde (Electron Microscopy Sciences, 15710) for 30 min, washed three times in 0.2% BSA in PBS (BSA/PBS 5 min each), permeabilized with 0.1% Triton X-100 (Thermo Fisher Scientific, HFH10) for 4 min, and blocked with 1% BSA/PBS for 1 h at room temperature. Primary antibodies were diluted at a 1:1,000 ratio in 1% BSA/PBS and incubated overnight at 4 °C. The following day, neurons were washed three times with PBS (5 min each) and incubated for 1 h in secondary antibodies diluted at a 1:2,000 ratio in 1% BSA/PBS. Neurons were then washed three times with PBS (5 min each) and counterstained with DAPI. Imaging was performed using the Operetta CLS high-content imaging system (Revvity/PerkinElmer) and analysed with Harmony software as previously described^33^.

For mitochondrial staining, hippocampal E16 C57BL/6J and *Ddhd2*^−/−^ neurons were incubated in high K^+^ or low K^+^ buffer for 5 min at 37 °C and 5% CO_2_, and then fixed with 4% paraformaldehyde in PBS for 20 min at room temperature. Cells were then washed thrice with PBS and thrice with 1% BSA in PBS (BSA/PBS), permeabilized with 0.1% Triton X-100 for 5 min, and then blocked with 1% BSA/PBS for 30 min. Primary mouse anti-synapsin 1 (Synaptic Systems, 106011; 1:1,000 dilution) and rabbit anti-TOMM20 (Abcam, ab186734; 1:1,000 dilution) antibodies were diluted in 1% BSA/PBS and incubated overnight at 4 °C. The following day, samples were washed thrice with PBS and then incubated for 1 h at room temperature with Alexa Fluor 488-conjugated Rat anti-mouse IgG (H + L; Invitrogen, A32723; 1:2,000 dilution) and Alexa Goat anti-Rabbit IgG (H + L; Invitrogen, A32733; 1:2,000 dilution), respectively (secondary antibodies protected from light). For the pulse-chase assay, hippocampal neurons were pulsed with high K^+^ supplemented with 1 µg ml^−1^ Alexa Fluor 555-conjugated recombinant CTxB (Invitrogen, C34776) or 50 µM tetramethylrhodamine-conjugated dextran (70,000 molecular weight; Invitrogen, 2113277) for 5 min. Cells were then washed three times with Neurobasal media and incubated in collected culture media for 25 min (chase). Neurons were fixed with 4% paraformaldehyde in PBS for 20 min, washed thrice with PBS and stained with DAPI (Sigma-Aldrich, D9542) for 1 h. Mounting of the samples was done in ProLong Gold Antifade Mountant (Thermo Fisher Scientific, P36930). Samples were imaged on a spinning-disk confocal (Marianas; 3I) consisting of an Axio Observer Z1 (Carl Zeiss) equipped with a CSU-W1 spinning-disk head (Yokogawa Corporation of America), an ORCA-Flash4.0 v2 sCMOS camera (Hamamatsu Photonics), a ×63/1.2-NA C-Apochromat objective and SlideBook 6.0 (3I). Images were acquired randomly. Quantification of MFI of dextran/CTxB per field of view was normalized by cell number (DAPI count) using Fiji/ImageJ (v2.14.0/1.54 f). Colocalization analysis was conducted using CellProfiler v4.2.8 (Broad Institute).

### Live-cell imaging and analysis

Hippocampal neurons from E16 C57BL/6J and *Ddhd2*^−/−^ embryos were seeded on glass-bottom dishes coated with 1 mg ml^−1^ PLL at cell densities of 30,000 cells per dish. For live-cell imaging, neurons were incubated with 200 nM MitoTracker Green FM (Invitrogen, M7514) for 15 min before imaging. Image acquisition was performed using a Zeiss C Plan-Apochromat ×63/1.4-NA oil-immersion objective on a confocal/two-photon laser-scanning microscope (LSM 980 NLO Airyscan 2, Carl Zeiss) built around an Axio Observer 7 body and equipped with an Airyscan 2 super-resolution detector, a 34-channel spectral photomultiplier tube (PMT) array, two internal GaAsP PMTs, a transmission PMT and two external GaAsP PMTs for non-descanned detection in two-photon microscopy, and controlled by Zen Blue software.

High-content mitochondrial assessments were done as previously described^33^. Briefly, total mitochondrial content was measured using staining with 200 nM MitoTracker Deep Red FM (Thermo Fisher Scientific, M22426) for 30 min, and ROS production with 400 nM MitoTracker Red CM-H_2_Xros (Thermo Fisher Scientific, M7513) staining for 30–45 min. Menadione crystalline (20 µM for 20–30 min; Merck, M5625) was used as a positive. Neurons were then rinsed and counterstained with Hoechst 33342, rinsed again and returned to the medium without phenol red for imaging. The Operetta CLS high-content analysis system (Revvity/PerkinElmer) was used for imaging. Unbiased automatic quantification was completed using Harmony software, with analysis pipelines modified from predefined Harmony algorithms. Briefly, nuclear regions were determined using Hoechst staining, with modifications as required for threshold, diameter and splitting sensitivity. Neuronal soma regions were calculated by subtracting the nuclear region from the total cell body, with thresholds set to restrict the cytoplasmic regions of interest to the perinuclear space. Border objects and irregular or dead cells were excluded. Spot detection algorithms were used to identify individual puncta of mitochondrial staining along neuronal processes. Generally, between 40 and 60 fields of view were acquired per well of the 96-well plate, capturing between 200,000 and 400,000 cells, with the Find Spots algorithm generally identifying around 400,000 to 700,000 puncta per well. Size, shape and fluorescence intensity parameters of selected objects were measured. Fluorescence intensity was calculated as a per-pixel average, and individual puncta intensities were normalized to the background intensity (corrected intensity = spot maximum / spot background). For live-cell confocal imaging, E16 hippocampal neurons were grown on glass-bottom dishes incubated with 200 nM MitoTracker Green FM (Invitrogen, M7514) for 15 min before imaging. Images were acquired using a Zeiss C Plan-Apochromat ×63/1.4-NA oil-immersion objective on an LSM 980 NLO Airyscan 2 confocal/two-photon microscope (Carl Zeiss) built on an Axio Observer 7 platform, equipped with an Airyscan 2 super-resolution detector, a 34-channel spectral PMT array, internal and external GaAsP PMTs and controlled by Zen Blue software.

### Western blotting

For western blotting, neurons were lysed in ice-cold lysis buffer (10 mM Tris-HCl pH 7.5, 150 mM NaCl, 0.5 mM EDTA (Merck, E4884), 0.5% NP-40, EDTA-free protease inhibitor, Merck, 4693159001), centrifuged at 15,800*g* for 15 min, and protein concentration was measured using the BCA assay (Thermo Fisher Scientific, 23225). Equal protein amounts (25 µg) were resolved on 4–20% precast polyacrylamide gels (Bio-Rad) at 100 V for 1 h, transferred to PVDF membranes (100 V, 90 min) and blocked with Intercept blocking buffer (LI-COR, 927-70001) for 1 h. Membranes were incubated overnight at 4 °C with primary antibodies against GFAP (Abcam, ab7260; 1:1,000 dilution), MAP-2 (Synaptic Systems, 188004; 1:1,000 dilution) and β-actin (Sigma-Aldrich, A5316; 1:1,000 dilution), followed by a 1-h incubation with secondary antibodies anti-mouse IR680 (926-68070; 1:5,000 dilution), anti-rabbit IR800 (926-32211; 1:5,000 dilution) and anti-guinea pig IR800 (926-32411; 1:5,000 dilution; all from LI-COR). Detection was performed using a LI-COR imaging system.

### Quantification of mtDNA copy number

mtDNA quantification was conducted using qPCR, as previously described^77^. Briefly, total DNA was extracted at DIV 21–22 from E16 primary neuronal cultures (treated with 4 µM Ara-C) derived from C57BL/6J and *Ddhd2*^−/−^ mice, with or without 1 µM or 10 µM M:P:S-CoA treatment for 48 h, using the PureLink Genomic DNA Mini Kit (Invitrogen, K182000). Following the manufacturer’s protocol, cells were resuspended in PBS and transferred to tubes containing proteinase K. After adding 10 ml of RNase, samples were vortexed and incubated at room temperature for 2 min. Next, 200 ml of PureLink Genomic Lysis/Binding Buffer was added, vortexed to ensure homogeneity, and incubated at 55 °C for 10 min to facilitate protein digestion. This was followed by the addition of 200 ml of 100% ethanol. DNA was then purified using a spin column-based centrifugation procedure. DNA concentration and purity were assessed using a NanoDrop One spectrophotometer (Thermo Fisher Scientific). The oligonucleotides mMitoF1 and mMitoR1 used to amplify mouse mtDNA, and the oligonucleotides mB2MF1 and mB2MR1 used to amplify nuclear DNA are listed in Supplementary Table 6. The qPCR was set up in 10 μl reactions with 5 μl of SensiFAST SYBR Green (Bioline), 0.25 μl of each primer and 1 μl of 1:100 diluted total DNA. qPCR was performed in a Rotor Gene Q Real-Time PCR system (Qiagen). The cycle conditions were 94 °C (5 min), followed by 40 cycles of two-step cycling, consisting of 94 °C (10 s) and 60 °C (30 s). Raw Ct values between three technical replicates were excluded if they had a difference of 0.5 or greater. The fold change in mtDNA copy number was calculated using the 2^−ΔΔCt^ method.

### Quantification of mtDNA damage

mtDNA damage was quantified using the LongAmp qPCR protocol, as previously described^78,79^. Briefly, total DNA extracted at DIV 21–22 from E16 neuronal cultures (+4 µM Ara-C) of C57BL/6J and *Ddhd2*^−/−^ ± 1 µM or 10 µM M:P:S-CoA treatment for 48 h. The oligonucleotides mtDNA F1 and mtDNA SR-1 used to amplify a 72-bp short amplicon from mouse mtDNA, and oligonucleotides mtDNA F1 and mtDNA LR-1 used to amplify 1,739-bp-long amplicons are listed in Supplementary Table 6. qPCR was set up in 10 μl reactions with 5 μl of SensiFAST SYBR Green (Bioline), 0.25 μl of each oligonucleotide and 1 μl of 1:100 diluted total DNA. qPCR was performed in a Rotor Gene Q Real-Time PCR system (Qiagen). The cycle conditions were 94 °C for 5 min, followed by 40 cycles of a two-step cycling protocol consisting of 94 °C for 10 s and 60 °C for 1 min. The mtDNA lesion frequency is calculated based on Poisson expression: -ln (AD/Ao), where AD represents the damaged amplicon and A0 represents the control amplicon, which is set to 1.

### Metabolic flux analysis in primary neurons

Metabolic fluxes in primary hippocampal neurons (DIV 19–22) were assessed in real time using the XFe96 Extracellular Flux Analyzer with Seahorse Wave software v2.6.1.56 (Seahorse Bioscience) as previously described^80^. Neurons were seeded at 25,000 cells per well on 1 mg ml^−1^ PLL-coated XFe96 plates, treated with 4 µM Ara-C on DIV 4 with subsequent treatments with etomoxir, and M-CoA, P-CoA and S-CoA as indicated, and assayed using the Seahorse XF Glycolysis Stress Test (Agilent, 103020-100) and XF Cell Mito Stress Test Kit (Agilent, 103010-100) per the manufacturer’s instructions. Before the assays, XF sensor cartridges were hydrated and calibrated with Seahorse Bioscience calibrant overnight at 37 °C incubator without CO_2_, and cell viability, confluence and morphology were confirmed via brightfield microscopy. All experiments were carried out at DIV 19–22, and the results were normalized to cell number. Data analysis to calculate basal and maximal respiration, ATP production, non-mitochondrial oxygen consumption, basal and maximal glycolysis, glycolytic reserve and non-glycolytic acidification was performed following the manufacturer’s instructions.

For ECAR measurements, neurons were equilibrated in XF assay medium (DMEM without phenol red, glucose, sodium bicarbonate or pyruvate; Agilent, 103575-100) for 45 min at 37 °C without CO_2_. Following instrumental calibration, glucose (10 mM), oligomycin (2 µM) and 2-DG (150 mM) were loaded into ports A–C, respectively. Before glucose injection, ECAR was measured over three cycles to establish the non-glycolytic acidification rate, after which glucose, oligomycin and 2-DG were sequentially injected onto plates, with ECAR measured over cycles of 1 min of mixing, 2 min of waiting and 3 min of measurement establishing basal glycolysis, maximal glycolytic capacity, glycolytic reserve and non-glycolytic acidification rates. For acute glycolysis stress, neurons were pre-equilibrated in DMEM lacking glucose and additives (Agilent, 103575-100), and then treated sequentially with etomoxir (40 µM), glucose (10 mM), oligomycin (2 µM) and 2-DG (150 mM) via ports A–D, respectively as described above.

For OCR measurements, neurons were incubated in XF assay medium (DMEM with 10 mM or 2 mM glucose, 2 mM glutamine, 1 mM pyruvate; Agilent, 103680-100) for 45 min at 37 °C without CO_2_. Following instrumental calibration, oligomycin (2 µM), FCCP (2.5 µM) and Rot/AA (1 µM each) were loaded into ports A–C, respectively. For β-oxidation assays, neurons were incubated in glucose-containing DMEM (Agilent, 103680-100) and treated with etomoxir (40 µM; or culture media as a control), oligomycin (1.5 µM), FCCP (2.5 µM) and Rot/AA (1 µM each) via ports A–D, respectively. Basal OCR was recorded over three measurement cycles before the sequential injection of modulators, and then the OCR was measured over cycles of 1 min of mixing, a 2-min wait and a 3-min measurement establishing basal respiration, maximal respiration, non-mitochondrial oxygen consumption and ATP production rates. For combined OCR/ECAR monitoring, neurons were incubated in full XF assay medium (Agilent, 103680-100), and baseline OCR and ECAR were recorded before injection of oligomycin (2 µM) and Rot/AA (1 µM each).

### RNA isolation, Illumina sequencing and analysis

Total RNA was extracted from NMRI mouse cortical neuron cultures at DIV 1 and DIV 20 using the ReliaPrep RNA Miniprep Kit (Promega, Z6010), followed by DNase I treatment (NEB, M0303S) and purification with RNAClean XP magnetic beads (Beckman Coulter, A63987). Strand-specific, polyA-selected libraries with ERCC spike-ins were prepared and deep sequenced by Azenta Life Sciences on an Illumina NovaSeq 2 × 150-bp run, yielding 22–51 million reads per sample. Reads were trimmed with Trimmomatic v0.36 and aligned to the *Mus musculus* GRCm38 reference genome using STAR aligner v.2.5.2b. Differential expression between DIV 20 and DIV 1 was analysed using DESeq2 with the Wald test.

### Quantitative mass spectrometry analysis of NMRI mouse cortical neurons

The proteins were precipitated on amine beads as previously described^81^. The precipitated proteins on beads were dissolved in 50 mM ammonium bicarbonate, reduced, alkylated and digested with trypsin (1:50 enzyme:protein ratio; Promega) at 37 °C overnight. The resulting peptide mixture was purified using the STAGE-TIP method with a C18 resin disk (3 M Empore) before the samples were analysed by a nanoLC–MS/MS using nanoElute coupled to timsTOF PRO2 (Bruker) with a 60-min separation gradient and a 25-cm Aurora C18 column. Mass spectrometry LC–MS/MS was performed using the Ultimate UHPLC system coupled to an Exploris 480 mass spectrometer with a FAIMS Pro interface (Thermo Fisher Scientific). Mass spectrometry raw files were submitted to MaxQuant software version 2.4.7.0 for protein identification and label-free quantification. Carbamidomethyl (C) was set as a fixed modification and acetyl (protein N-term), carbamyl (N-term) and oxidation (M) were set as variable modifications. A first search peptide tolerance of 20 ppm and a main search error of 10 ppm were used. Trypsin without the proline restriction enzyme option was used, with two allowed miscleavages. The minimal unique + razor peptides number was set to 1, and the allowed false discovery rate was 0.01 (1%) for peptide and protein identification. Label-free quantification was used with default settings. UniProt database with ‘mouse’ entries (2020) was used for the database searches. Additional data filtering and statistical analysis was done using Perseus version 1.6.1.5. using normalized intensities (LFQ).

### Quantitative mass spectrometry analysis of C57BL/6J and *Ddhd2*^−/−^ cortical neurons and neuron–glia cultures

The LC–MS/MS identification and quantification of the digested peptides from primary cortical neurons (+4 µM Ara-C) or neuron–glia (no Ara-C) cultures of E16 C57BL/6J and *Ddhd2*^−/−^ ± 1 µM S-CoA, 1 µM M:P:S-CoA or 1 µM M-CoA, for 48 h, were performed at DIV 21–22 using an S-Trap (Protifi) Micro Spin Column Digestion protocol. Briefly, samples were solubilized by adding 50 ml of S-Trap lysis buffer (10% sodium dodecyl sulfate (SDS; Sigma-Aldrich, 436143) in 100 mM Tris, pH 8.0 and 1× cOmplete (EDTA-free protease inhibitor cocktail, Sigma-Aldrich, 11836170001) to 50 ml of sample, before reducing by adding 20 mM of dithiothreitol (Sigma-Aldrich, D9760) and heating at 70 °C for 60 min. Cysteine residues were alkylated to prevent disulfide bond reformation using 40 mM iodoacetamide (Sigma-Aldrich, A3221) for 30 min at room temperature in the dark. Next, 2.5 ml of 12% phosphoric acid (Sigma-Aldrich, 695017) was added, followed by 165 ml of S-Trap binding buffer (90% methanol (Sigma-Aldrich, 1424109) in 100 mM Tris) to the acidified lysate. The sample mix was then centrifuged through the S-Trap column at 4,000*g* for 1 min followed by three washes with 150 ml S-Trap binding buffer, with 4,000*g* centrifugation between each wash. Peptide digestion was initiated by adding 25 ml of 50 mM ammonium bicarbonate buffer (pH 8; Sigma-Aldrich, 09830) containing 2 µg trypsin (Sequencing Grade Modified Trypsin, Promega, V5117) directly on top of the column and incubating overnight at 37 °C. Peptides were eluted by three successive aliquots of 40 ml of 5%, 50% and 75% acetonitrile (Sigma-Aldrich, PHR1551) in 0.1% formic acid (Sigma-Aldrich, 399388), respectively. Eluted peptides were dried down using a vacuum concentrator (Concentrator Plus, Eppendorf). Samples were redissolved in 20 ml of 5% acetonitrile (aq) and 2 ml was injected to a trap column (Thermo Fisher Scientific, 22 mm × 300 µm, 5 µm, C18) at a flow rate of 10 ml min^−1^. Following 3 min of washing, the trap column was switched in-line with a resolving column (Water nanoEase 100 mm × 150 µm, 1.8 µm, 100 Å). The samples were eluted by a gradient that was held constant at 8% for 4 min, then was increased linearly to 24% at 47 min, to 40% at 53 min and to 95% at 57 min. The gradient held constant for 1 min, before returning to the start condition at 8% over 1 min. LC–MS/MS was performed using the Ultimate UHPLC system as described above. The FAIMS compensation voltages were −45 V and −65 V. The electrospray voltage was 2.2 kV in positive-ion mode, and the ion transfer tube temperature was 295 °C. Full MS scans were acquired in the Orbitrap mass analyser over the range of *m/z* 340–1,110 with a mass resolution of 120,000. The automatic gain control target value was set at ‘Standard’, and the maximum accumulation time was ‘Auto’ for the mass spectrometry. The MS/MS ions were measured in 6 windows from 350–470 *m/z*, in 18 windows from mass 465–645 *m/z* and 5 windows from 640–1,100 *m/z* with an overlap of 1 *m/z* and quadrupole isolation mode. Analysis of data was performed using Spectronaut against a reference proteome with a *q*-value cut-off of 0.05.

### Label-free mass spectrometry of C57BL/6J and *Ddhd2*^−/−^ neuron–glia and neuronal cultures

For the processing and analysis of C57BL/6J and *Ddhd2*^−/−^ neuron–glia cultures (no Ara-C), the standard non-normalized output of Spectronaut (BGS Factory Report text file) was imported into R version 4.05 for further processing using a modified method described previously^82^. For proteins that mapped to multiple annotations, the ‘best annotation’ method previously described was used^82^. Each sample group was permitted to have up to one missing value (that is, an intensity not reported in one of the three individual replicates). Protein intensities of the parent group protein ‘PG.ProteinGroups’ were then log_2_ transformed and globally normalized using the quantile normalization method^83^. Missing values were assumed to be missing at random and imputed using the *k*-nearest-neighbour averaging method^84^. Following this, unwanted sources of technical variation were removed by surrogate variable analysis^85^. Sample clustering was confirmed using unsupervised principal component analysis and hierarchical clustering. Principle component analysis was performed using the first two principal components with singular value decomposition, as implemented in pcaMethods version 1.82.0 and hierarchical clustering using ‘ward’ method for clustering distance ‘euclidean’. For hierarchical clustering, probabilities of clustering were determining using 100 bootstrap replications, as implemented in pvcluster version 2.2-0, and probabilities of the branch positions shown in the plot, together with the probabilities of clusters (red boxes). For differential protein abundance, generalized linear modelling with Bayes shrinkage as implemented in limma version 3.46.0 was performed, and proteins were considered differentially abundant at a corrected *P* value of 0.05 unless specified otherwise (adjusted for false discovery rate using the Benjamini and Hochberg method)^86^.

For the processing and analysis of C57BL/6J and *Ddhd2*^−/−^ neuronal cultures (+Ara-C), the standard non-normalized output from Spectronaut (BGS Factory Report text file) was imported into R (version 4.4.2, 2024-10-31 ucrt) for downstream statistical analysis. Protein intensity values were log_2_ transformed and median normalized. Proteins were retained if quantified in at least 80% of samples within any experimental group. Missing values were imputed using the QRILC method (quantile regression imputation of left-censored data), as implemented in the imputeLCMD R package (10.1021/acs.jproteome.5b00981). To account for batch effects and other sources of unwanted technical variation, surrogate variable analysis was applied using the sva package (10.1093/bioinformatics/bts034). Sample relationships and group separability were assessed by principal component analysis and hierarchical clustering using Euclidean distance and Ward’s method. Differential protein abundance analysis was conducted using linear modelling with empirical Bayes shrinkage, implemented via the limma package (10.1093/nar/gkv007).

### Gene-set enrichment analyses of C57BL/6J and *Ddhd2*^−/−^ neuron–glia and neuronal cultures

For C57BL/6J and *Ddhd2*^−/−^ neuron–glia cultures (no Ara-C), heat maps for proteins belonging to a gene set (GO term) were independently generated by extracting all gene symbols from ‘org.Mm.eg.db’ version 3.1.2 that matched a specific GO term of interest. This was then filtered by proteins that were differentially abundant between *Ddhd2*^−/−^ myristic acid treated versus *Ddhd2*^−/−^ (comparison of interest). The *z*-score of the protein intensities across all samples (row-wise) was then calculated across samples and plot as a heat map using the pheatmap version 1.0.12. Clustering distance was ‘Euclidean’ using a linkage method.

For C57BL/6J and *Ddhd2*^−/−^ neuronal cultures (+Ara-C), GO enrichment analysis was performed separately for the Biological Process (BP), Cellular Component (CC) and Molecular Function (MF) ontologies using the clusterProfiler package (v 4.14.6; 10.1089/omi.2011.0118), with mouse gene annotations provided by the org.Mm.eg.db database. Significantly enriched GO terms were identified using a *P-*value cut-off of 0.05. To reduce redundancy among enriched GO terms, semantic similarity-based clustering was performed using the rrvgo package (10.17912/micropub.biology.000811). Pairwise GO term similarity was computed using the ‘Rel’ method from the GOSemSim package (10.1093/bioinformatics/btq064), and terms were grouped using a similarity threshold of 0.8. To illustrate protein expression patterns associated with specific subcellular compartments, heat maps were generated based on proteins annotated to representative GO and R-HSA terms listed in Supplementary Table 7. For each term, proteins were extracted from GO enrichment results, and their log_2_-transformed, batch-corrected intensities were *z*-score normalized across samples (row-wise) to highlight relative abundance shifts across conditions. Heat maps were generated using the pheatmap R package (v 1.0.12) with hierarchical clustering (Euclidean distance, complete linkage).

### Ultrastructural analysis of cultured neurons using TEM

Transmission electron microscopy (TEM) was performed on E16 C57BL/6J and *Ddhd2*^−/−^ (±1 µM fatty acyl-CoA treatments for 48 h) neuronal cultures (+4 µM Ara-C) and imaged at DIV 21–22. For presynaptic and mitochondrial analysis, neurons were incubated in low K^+^ buffer (resting) or stimulated for 5 min in high K^+^ buffer, and then fixed in 2% glutaraldehyde (Electron Microscopy Sciences, 16210) in 0.1 M sodium cacodylate buffer (pH 7.4; Sigma-Aldrich, C0250) for 20 min at room temperature. After two 3-min washes in 0.1 M sodium cacodylate, samples were contrasted with 1% osmium tetroxide (EMS, 19100) and 1.5% potassium ferrocyanide (EMS, 25154-10) in the same buffer, followed by 2% uranyl acetate (EMS, 22400). Samples were dehydrated in ethanol series and embedded in LX112 resin (Ladd, 21210) using a BioWave system (Pelco). For peroxidase cytochemistry, neurons were either stimulated in high K^+^ buffer with 10 mg ml^−1^ HRP at 37 °C and 5% CO_2_ for 5 min or incubated in low K^+^ buffer with 1 mg ml^−1^ HRP and stimulated at 50 Hz (300 APs, 6 s), then washed and incubated for 10 min or 30 min at 37 °C and 5% CO_2_ (that is, chase). Fixation was done using 2% glutaraldehyde and 2% paraformaldehyde in 0.1 M sodium cacodylate buffer (pH 7.4) for 20 min at room temperature, followed by two washes with buffer. After three 5-min washes in 50 mM Tris buffer (pH 7.6), neurons were stained with 3,3′-diaminobenzidine (Sigma-Aldrich, D5905) containing H2_2_O_2_ (Sigma-Aldrich, 7722-84-1) for 30 min at room temperature. Samples were then contrasted with 1% osmium tetroxide and 2% uranyl acetate (Electron Microscopy Sciences, 22400), dehydrated and embedded in LX112 resin using a BioWave system (Pelco). Ultrathin sections (80–90 nm) were cut on a Leica UC6FCS ultramicrotome and imaged with JEOL 1101 and 1400 TEMs equipped with an Olympus Morada CCD camera. The numbers of synaptic vesicles, endosomes and glycogen granules were counted from electron micrographs using Adobe Photoshop (Adobe, 22.4.3 release) Count Tool and related to the presynaptic area (µm^2^), which are manually segmented and measured using the ImageJ/Fiji (https://imagej.nih.gov/ij/) measurement tool. The same tool was also used to measure the size of endosomes, presynapses, mitochondria and HRP-stained endosomes (µm^2^). Vesicles with sectional area ≤0.003 µm^2^ are classified as synaptic vesicles, and those >0.003 µm^2^ as endosomes. For quantification, 95,150 synaptic vesicles and 2,475 endosomes were counted in total. Presynapses are identified as rounded structures enriched with synaptic vesicles, typically positioned adjacent to PSDs and connected to axons. Axons were defined as elongated structures often containing microtubules, presynaptic connections, and having a diameter >200 nm, and soma as the region of a neuron that includes the cell body of cell soma and dendrite(s), but excludes the axon, and often has a visible nucleus and PSDs. The subcellular location of mitochondria was calculated by counting the number of mitochondrial cross-sections per presynapse. It is worth noting that this quantification does not present the absolute presynaptic numbers of presynaptic mitochondria, as the electron microscopy sections only capture a thin section of the synapse (for example, elongated mitochondria could span the sections multiple times), but the quantification rather reflects the overall mitochondrial presence in the presynapses.

### Fluorescence imaging of vGlut1-pHluorin

To monitor synaptic vesicle recycling, we utilized the pH-sensitive green fluorescent protein pHluorin fused with vesicular glutamate transporter 1, that is, vGlut1-pH^51^. The construct functions as a reporter of synaptic vesicle exocytosis and endocytosis and vesicular reacidification. Its fluorescence intensity is quenched within the acidic vesicle lumen and subsequently dequenched following synaptic vesicle fusion^52^. Live hippocampal C57BL/6J and *Ddhd2*^−/−^ neurons transiently expressing vGlut1-pHluorin were mounted in an imaging chamber with field stimulation electrodes (RC-21BRFS; Warner Instruments) and continuously perfused with imaging buffer (119 mM NaCl, 2.5 mM KCl, 2 mM CaCl_2_, 2 mM MgCl_2_, 25 mM HEPES, 30 mM d-glucose, pH 7.4) supplemented with 10 μM NBQX (Abcam, ab120046) and 50 μM DL-APV (Abcam, ab120271). All experiments were performed at 35 °C. The imaging solution was kept constant at 35 °C using an in-line solution heater (SH-27B; Warner Instruments). Before stimulation, the basal fluorescence intensity of the reporter vGlut1-pHluorin was recorded in each C57BL/6J and *Ddhd2*^−/−^ acquisition. The regions of interest were chosen randomly. Neurons expressing vGlut1-pHluorin were challenged with a train of 300 action potentials delivered at 50 Hz and 6 s (100 mA and 1-ms pulse width) and imaged at 0.5 Hz (2 × 2 binning) through a ×40 (1.4-NA) oil objective using an inverted Zeiss Axio Observer Z1 epifluorescence microscope equipped with an Andor Luca R EMCCD camera. At the end of each imaging acquisition, neurons were perfused with an alkaline imaging buffer (50 mM NH_4_Cl, Sigma-Aldrich, 213330, substituted for 50 mM NaCl) to reveal total pHluorin expression. Equal-sized regions of interest were placed over nerve terminals to measure the pHluorin fluorescence elicited by stimulation over time using the Time Series Analyzer plugin in Fiji software (National Institutes of Health). Activity-dependent changes in fluorescence (Δ*F/F*_0_) were normalized to the respective peak heights from the train of stimuli (to calculate the rate of endocytosis) or to the total amount of fluorescence present after alkaline treatment (to calculate the exocytosis amplitude). The rapid increase in the vGlut1-pHluorin fluorescence signal following stimulation represents fusion of synaptic vesicles to the plasma membrane, while the compensatory retrieval of membranes via endocytosis results in the re-internalization of vGlut1-pHluorin molecules into acidic synaptic vesicles, which corresponds to a gradual decrease in the fluorescence signal of the reporter. The rate of fluorescence decline is described by the endocytosis time constant (τ value; that is, the time required for the fluorescence signal to reach zero if the rate of the decline was linear), which was calculated by fitting the decay phase for each trace to a single exponential function^52^.

### Synaptic ATP sensors

Synaptic ATP levels were investigated using a genetically encoded, quantitative ATP sensor that enables real-time measurement of ATP dynamics at the synapse as previously described^34^. pAAV.hSynapsin.(cyto).iATPSnFR2.A95K.HaloTag^34^ (Addgene plasmid, 209664) and hSynapsin.cpSFGFP.HALOTag-JF549^34^ (Addgene plasmid, 209666) were a gift from T. Brown and the HHMI-JRC Tool Translation Team. AAV9-mediated expression of these chimeric constructs was induced in C57BL/6J and *Ddhd2*^−/−^ neurons at DIV 9 (MOI 10, equivalent of 2,500 viral genomes per neuron). Neurons were incubated with 200 nM Janelia Fluor 549 HaloTag Ligand (Promega, GA1110) for 15 min before imaging at 37 °C and 5% CO_2_. Simultaneous imaging of HaloTag-JF549 and iATPSnFR2 or cpSFGFP was performed at DIV 19 using a Roper Scientific iLas^2^ Ring-TIRF microscope with a CFI Apo ×100/1.49-NA oil-immersion objective (Nikon Instruments) and additional magnification of ×1.5, two Evolve 512 Delta EMCCD cameras (Photometrics) mounted on a TwinCam LS Image Splitter (Cairn Research) for simultaneous dual-channel imaging, a Perfect Focus System (Nikon), an iLas^2^ double-laser illuminator (Roper Scientific) for 360° TIRF illumination, using 488-nm and 561-nm lasers (150 mW, Cobolt Jive). Visualization was done at 50 Hz by acquiring 1,000 frames per condition by image streaming with a 20-ms exposure time. Synaptic ATP levels were quantified as the green:red fluorescence ratio in resting, high K⁺ stimulated and 2-DG-treated conditions (150 mM, 15 min) in manually segmented synapses in each condition. A green:red ratio was then plotted as a time course for each condition, showing averaged MFI ± s.e.m. normalized to the average of C57BL/6J *t*_1_ in hSynapsin.iATPSnFR2.HALOTag-JF549 acquisitions. The maximum green:red fluorescence intensity change recorded in each synapse was normalized to the average of C57BL/6J in each experiment and the numbers are plotted as paired graphs.

For the analysis of HSP54 mutant rescue with fatty acyl-CoA treatment, E16 *Ddhd2*^−/−^ neurons were infected with lentiviral control, Fuw-DDHD2^WT^-myc, Fuw-DDHD2^D660H^-myc, Fuw-DDHD2^S351A^-myc or Fuw-DDHD2^W103R^-myc at DIV 4, and the expression of DDHD2 in *Ddhd2*^−/−^ neurons was analysed at DIV 14 using anti-DDHD2 (Proteintech, 25203-1-AP; 1:1,000 dilution) in a standard western blot protocol, in comparison to endogenous Ddhd2 in C57BL/6J (and *Ddhd2*^−/−^ neurons). Mouse anti-β-actin (Sigma-Aldrich, A5316; 1:5,000 dilution) was used as the loading control. To assess the efficacy of M:P:S-CoA treatment in the HSP54 background, *Ddhd2*^−/−^ neurons were lentivirally induced with Fuw-DDHD2^WT^-myc or Fuw-DDD2^W103R^-myc at DIV 3 and, along with C57BL/6J, infected with AAV9 at DIV 7 to transiently express the chimeric construct hSynapsin.iATPSnFR2.HALOTag-JF549 (ATP sensor). Around 1 µM M:P:S-CoA for 48-h treatment was then carried out in *Ddhd2*^−/−^ neurons expressing Fuw-DDHD2^W103R^-myc before imaging at DIV 14–16 (M:P:S-CoA group). Imaging and quantification were done as described above.

### Single-molecule imaging

For sptPALM^87^ and universal point accumulation imaging in nanoscale topography^53,88^, hippocampal neuron cultures of E16 C57BL/6J and *Ddhd2*^−/−^ (±1 µM fatty acyl-CoA treatments for 48 h as indicated) were transfected at DIV 14 with pmEOS2-C1-ERGIC53 (ERGIC53-mEos2) or VAMP2–pHluorin (a gift from V. Haucke, Leibniz-Forschungsinstitut für Molekulare Pharmakologie Berlin), respectively, using Lipofectamine 2000 (Thermo Fisher Scientific, 11668027) according to the manufacturer’s instructions and imaged at DIV 21–22. For sptPALM, time-lapse movies (10,000 frames) were acquired at 50 Hz using a Roper Scientific Ring-TIRF microscope equipped with an iLas^2^ double-laser illuminator (Roper Scientific), a Nikon CFI Apo TIRF ×100/1.49-NA oil-immersion objective (Nikon Instruments) and a Perfect Focus System (Nikon Instruments). Imaging was performed using two Evolve512 delta EMCCD cameras (Photometrics) mounted on a TwinCam LS Image Splitter (Cairn Research), a quadruple beam splitter (ZT405/488/561/647rpc, Chroma Technology) and a QUAD emission filter (ZET405/488/561/640 m, Chroma Technology) and Metamorph software (MetaMorph Microscopy Automation and Image Analysis Software, v7.7.8; Molecular Devices) as described previously^48^. Imaging was done on regions of interests that contained neurons expressing the construct. The 405-nm laser was used to photo-convert ERGIC53-mEos2, and the 561-nm laser was used simultaneously for excitation and bleaching of the resulting photo-converted single molecules. To isolate the mEos2 signal from autofluorescence and background signals, a double-beam splitter (LF488/561-A-000, Semrock) and a double-band emitter (FF01-523/610-25, Semrock) were used. Imaging was done on regions of interests that contained neurons expressing the construct. To spatially distinguish and temporally separate the stochastically activated molecules during acquisition, the 405-nm laser was used between 1.5% and 5% of the initial laser power (100 mW Vortran Laser Technology), and the 561-nm laser was used at 80% of the initial laser power (150 mW, Cobolt Jive). To image VAMP2–pHluorin mobility in resting and stimulated conditions, hippocampal neurons expressing VAMP2–pHluorin were washed once with low K^+^ buffer and then imaged in low K^+^ buffer containing 100 pM anti-GFP Atto565N nanobodies (Synaptic Systems, N0301-At565) acquiring time-lapse movies (10,000 frames) at 50 Hz using the TIRF system described above. Unbound nanobodies were then washed off by washing thrice with low K^+^ buffer and subsequently imaged in high K^+^ buffer containing 100 pM anti-GFP Atto647N (Synaptic Systems, N0301-At647N) nanobodies for 10,000 frames at 50 Hz.

PALMTracer^89–91^ in Metamorph software was used to obtain the MSD and diffusion coefficient (*D*; μm^2^ s^−1^) values for single-molecule tracks. Tracks shorter than eight frames were excluded from the analysis to minimize non-specific background. The Log_10_*D* immobile and mobile fraction distributions were calculated as previously described^92^, setting the displacement threshold to 0.03 μm^2^ s^−1^ (that is, log_10_*D* = −1.45 when *D* = μm^2^ s^−1^). The mobile-to-immobile ratio was determined based on the frequency distribution of the diffusion coefficients (Log_10_*D*) of immobile (log_10_*D* ≤ −1.45) and mobile (log_10_*D* > −1.45) molecules (the immobile fraction of molecules represents VAMP2 and ERGIC53 molecules for which the displacement within FOUR frames was below the spatial detection limit of our methods, 106 nm). The area under the MSD curve was calculated in Prism 9 for macOS version 9.1.1 (GraphPad). The super-resolved image colour coding was done as previously described^71^ using ImageJ/Fiji (2.0.0-rc-43/1.50e; National Institutes of Health, https://imagej.nih.gov/ij/), with each coloured pixel in the average intensity maps indicating the localization of an individual molecule (bar: 8 to 0, high to low density), the colour-coded pixels in the average diffusion coefficient map presenting an average value for each single-molecule track at the site of localization (bar: log_10_ 1 to −5, high to low mobility), and the colour coding of the track maps representing the detection time point (bar: 0–10,000 frame acquisition) during acquisition.

### EPR measurements

To assess membrane fluidity^54^, the following nitroxyl radical SLFA probes were used: Spin-labelled 5-doxyl-stearic acid (5-DSA, 253618) and 16-doxyl-stearic acid (16-DSA, 253596) both from Sigma-Aldrich. 5-DSA was used to determine the local fluidity near the protein–aqueous interface, and 16-DSA was used to assess the fluidity near the hydrophobic protein cores. C57BL/6J and *Ddhd2*^−/−^ neurons (±1 µM M:P:S-CoA for 48 h), and neurosecretory PC12 cells at resting and stimulatory conditions, were labelled 0.02 M methanol-containing solutions of 5-DSA or 16-DSA and loaded into the capillary tubes to measure the EPR spectra at 37 °C using a Bruker E540 Benchtop Magnettech MiniScope MS5000 spectrometer. Instrumental parameters for these measurements were: microwave frequency, 9.4786 GHz; microwave power, 5.024 mW (16 dB attenuation of 200 mW source); modulation frequency, 100 kHz; modulation amplitude, 0.12 mT (16-DSA) or 0.3 mT (5-DSA). Resonances occur in the approximate, and magnetic field range 332346–342356 mT. The number of scans varied depending upon the required signal-to-noise ratio; each scan was 60 s, and the measurement time was typically 5 to 10 min. Each spectrum is an average of 5–10 scans with scan time of 60 s.

### Quantification and statistical analysis

Statistical tests were conducted in GraphPad Prism 9 for macOS version 9.1.1. No statistical methods were used to pre-determine sample sizes, but our sample sizes are similar to those reported in previous publications^2,12^. The normality of the data was tested with the GraphPad Kolmogorov–Smirnov test, and nonparametric tests and parametric tests were used to compare two independent groups (two-tailed Mann–Whitney test or two-tailed *t*-test) and multiple groups (ordinary one-way ANOVA Sidak’s, Tukey’s, Dunnett’s and Kruskal–Wallis multiple-comparison tests). If normality could not be determined, it was assumed. For Seahorse measurements, the ROUT method for outlier detection (Q 1%) function was used in GraphPad, and negative values arising from cell density that is too low were removed. No other data were excluded from analysis. Measurements were taken from distinct samples, unless otherwise stated, and all data are presented as the average ± s.e.m. Data points are independent biological replicates, except for Figs. 3g, 4 and 8 where the dots present measurements from individual synapses. Neurons for microscopy were selected from random field of views. The identified HSP54 cases show that the condition is similarly observed in both males (21 cases) and females (27 cases; Supplementary Table 3), and therefore mixed embryonic cultures were used (that is, sex was not determined) except for brain lysates that were from female brains. The investigators were not blinded to the experimental conditions during data collection and analysis. Specific statistical tests for each experiment and the exact *P* values are described in the figure legends or provided in figures. *P* values of less than 0.05 were considered significant.

### Reporting summary

Further information on research design is available in the Nature Portfolio Reporting Summary linked to this article.

## Supplementary information

Reporting Summary

Supplementary Video 1 Mitochondrial dynamics in cultured C57BL/6J hippocampal neurons. Live-cell imaging of cultured hippocampal C57BL/6J neurons stained with MitoTracker Green FM. The video shows maximum projected confocal stacks acquired at one frame every 8 s. The playback is five frames per second.

Supplementary Video 2 Mitochondrial dynamics in cultured *Ddhd2*^−/−^ hippocampal neurons. Live-cell imaging of cultured hippocampal *Ddhd2*^−/−^ neurons stained with MitoTracker Green FM. The video shows maximum projected confocal stacks acquired at one frame every 8 s. The playback is five frames per second.

Supplementary Table 1 mRNA expression analysis of cortical neurons. The table shows mRNA expression analysis of cortical neurons in wild-type NMRI mice.

Supplementary Table 2 Comparative proteomic analysis of cortical neurons. The table shows comparative proteomics analysis of wild-type E16 NMRI mice analysed at DIV 1 and DIV 20.

Supplementary Table 3 Human *DDHD2* mutations causing HSP54. The table shows the reported human mutations of *DDHD2* and associated symptoms of individuals with HSP54, and whether the DDHD2 mRNA and protein product levels were reported^6,11,13–15,37–48^. See also Extended Data Fig. 5.

Supplementary Table 4 Proteomic analysis of hippocampal neuronal cultures. The table shows proteomic analysis of hippocampal neurons (+Ara-C) from C57BL/6J, *Ddhd2*^−/−^ and *Ddhd2*^−/−^ supplemented with 1 µM S-CoA or M:P:S-CoA for 48 h and analysed at DIV 21–22.

Supplementary Table 5 Proteomic analysis of neuron–glia cultures. The table shows proteomic analysis of neuron–glia cultures from C57BL/6J, *Ddhd2*^−/−^ and *Ddhd2*^−/−^ supplemented with 1 µM M-CoA for 48 h and analysed at DIV 21–22.

Supplementary Table 6 Primers. The table shows primers used for cloning of FUW-DDHD2-myc and pmEOS2-C1-ERGIC53.

Supplementary Table 7 GO and R-HAS codes. The table contains GO and R-HAS codes used for proteomics heat maps in this study.

## Source data

Source Data Fig. 4i Uncropped western blot images of Fig. 4i.

Source Data Extended Fig. 1b Uncropped western blot image of Extended Data Fig. 1b.

## Data Availability

This study did not generate new unique reagents. The constructs created in this study have been deposited to Addgene. All original data created in this study and shown in Figs. 1–8 have been uploaded to The University of Queensland RDM 10.48610/0908106. Source data are provided with this paper. All other data that support the findings of this study are available from the corresponding author upon reasonable request.

## Code availability

All analysis code used for preprocessing, normalization, batch correction, differential expression, enrichment and visualization of the proteomics data is deposited in GitHub via https://github.com/bongrita/DDHD2_proteomics/.
