# Supplementary material for: DDHD2 provides a flux of saturated fatty acids for neuronal energy and function
Source: Nat Metab. 2025 Sep 30;7(10):2117–41. doi: 10.1038/s42255-025-01367-x (PMC12552131; doi:10.1038/s42255-025-01367-x)
Supplement: Supplementary file 1 — Reporting Summary [file 42255_2025_1367_MOESM1_ESM.pdf]

nature portfolio

Corresponding author(s): Merja Joensuu, NATMETAB-A240611980A

Last updated by author(s): 2025.05.01

Reporting Summary

Nature Portfolio wishes to improve the reproducibility of the work that we publish. This form provides structure for consistency and transparency in reporting. For further information on Nature Portfolio policies, see our Editorial Policies and the Editorial Policy Checklist.

Statistics

For all statistical analyses, confirm that the following items are present in the figure legend, table legend, main text, or Methods section.

n/a	Confirmed	
☐	☒	The exact sample size (*n*) for each experimental group/condition, given as a discrete number and unit of measurement
☐	☒	A statement on whether measurements were taken from distinct samples or whether the same sample was measured repeatedly
☐	☒	The statistical test(s) used AND whether they are one- or two-sided<br>*Only common tests should be described solely by name; describe more complex techniques in the Methods section.*
☐	☒	A description of all covariates tested
☐	☒	A description of any assumptions or corrections, such as tests of normality and adjustment for multiple comparisons
☐	☒	A full description of the statistical parameters including central tendency (e.g. means) or other basic estimates (e.g. regression coefficient) AND variation (e.g. standard deviation) or associated estimates of uncertainty (e.g. confidence intervals)
☐	☒	For null hypothesis testing, the test statistic (e.g. *F*, *t*, *r*) with confidence intervals, effect sizes, degrees of freedom and *P* value noted<br>*Give P values as exact values whenever suitable.*
☒	☐	For Bayesian analysis, information on the choice of priors and Markov chain Monte Carlo settings
☒	☐	For hierarchical and complex designs, identification of the appropriate level for tests and full reporting of outcomes
☒	☐	Estimates of effect sizes (e.g. Cohen's *d*, Pearson's *r*), indicating how they were calculated

Our web collection on statistics for biologists contains articles on many of the points above.

Software and code

Policy information about availability of computer code

Data collection	n/a
Data analysis	All softwares and codes used in the paper are published and either open source or commercially available. All have been described in materials and methods and links to websites or respective publication cited.

For manuscripts utilizing custom algorithms or software that are central to the research but not yet described in published literature, software must be made available to editors and reviewers. We strongly encourage code deposition in a community repository (e.g. GitHub). See the Nature Portfolio guidelines for submitting code & software for further information.

Data

Policy information about availability of data

All manuscripts must include a data availability statement. This statement should provide the following information, where applicable:

- Accession codes, unique identifiers, or web links for publicly available datasets
- A description of any restrictions on data availability
- For clinical datasets or third party data, please ensure that the statement adheres to our policy

All original data generated in this study and presented in the figures and supplementary figures and files will be uploaded upon acceptance of the manuscript in the University of Queensland on-line Research Data Management (RDM) system and the web link to the data will be provided in the publication. The data will be freely available for research purposes. No clinical datasets or third party data are included in our study.

Research involving human participants, their data, or biological material

Policy information about studies with human participants or human data. See also policy information about sex, gender (identity/presentation), and sexual orientation and race, ethnicity and racism.

Reporting on sex and gender	n/a
Reporting on race, ethnicity, or other socially relevant groupings	n/a
Population characteristics	n/a
Recruitment	n/a
Ethics oversight	n/a

Note that full information on the approval of the study protocol must also be provided in the manuscript.

Field-specific reporting

Please select the one below that is the best fit for your research. If you are not sure, read the appropriate sections before making your selection.

☒ Life sciences ☐ Behavioural & social sciences ☐ Ecological, evolutionary & environmental sciences

For a reference copy of the document with all sections, see nature.com/documents/nr-reporting-summary-flat.pdf

Life sciences study design

All studies must disclose on these points even when the disclosure is negative.

Sample size	Each experiment with staistical testing include 3-5 independent biological cell culture preparations from distinct biological sources (i.e., different animals). The choice of this number of repetitions was based on minimizing the use of animals while obtaining data that were statistically significantly different between controls and target samples with a confidence of at least 95%, as well as based on availability of knockout embryos. No statistical methods were used to pre-determine sample sizes, but our sample sizes are similar to those reported in previous publications. Normality of data distribution was tested for each experiment, and when negative, a more stringent non parametric multiple comparison test was used.
Data exclusions	For OCR measurements, Identify outliers, ROUT method (Q 1%) function was used in GraphPad and negative reading were removed. No other data were excluded from analysis.
Replication	All experiments were reproducible and all the raw data of each experiment will be provided at University of Queensland online RDM with a freely accessible link. The proteomics data are included as tables in the manuscript files.
Randomization	All neuronal/glial cultures were obtained from mixed embryos (no selection of particular embryos). For fluorescence imaging, we either used automated imaging (fully automated scanning of entire wells in multiplex plates, described in materials and methods), or for neuronal imaging of mitochondria, ERGIC, ATP sensor in synapses, super-resolution imaging of ERGIC53-mEos2 and Vamp2pHluorin, the selection of soma and synapses of cells was based on sufficient fluorescence signal and regular morphology of the neurons, soma and synapse. Multiple areas on each imaged dish or coverslip were acquired and the same criterion was used for all samples. For TEM analysis, the entire thin sections were visually scanned at the electron microscope and images acquired every time the organelle of interest or HRP signal was identified (e.g., ERGIC, Golgi, mitochondria, or synapses).
Blinding	n/a

Reporting for specific materials, systems and methods

We require information from authors about some types of materials, experimental systems and methods used in many studies. Here, indicate whether each material, system or method listed is relevant to your study. If you are not sure if a list item applies to your research, read the appropriate section before selecting a response.

Materials & experimental systems

n/a	Involved in the study
☐	☒ Antibodies
☐	☒ Eukaryotic cell lines
☒	☐ Palaeontology and archaeology
☐	☒ Animals and other organisms
☒	☐ Clinical data
☒	☐ Dual use research of concern
☒	☐ Plants

Methods

n/a	Involved in the study
☒	☐ ChIP-seq
☒	☐ Flow cytometry
☒	☐ MRI-based neuroimaging

Antibodies

Antibodies used

All antibodies used in the study are commercial. For immunofluorescence: GFAP (Abcam, ab7260, RRID:AB_305808), MAP-2 (Synaptic Systems, Cat. no. 188004; RRID:AB_2138181), Synapsin-1(Synaptic Systems, 106011, RRID:AB_2619772), TOMM20 (Abcam, ab186734, RRID:AB_2716623), Alexa Fluor 647 anti-rabbit IgG (ThermoFischer Scientific, A-21245, RRID:AB_2535813), Alexa Fluor 488 anti-mouse IgG (ThermoFischer Scientific, A-11001, RRID:AB_2633275) anti-Rabbit IgG Alexa Fluor Plus 647 (ThermoFischer Scientific, A32733, RRID:AB_2633282). For western blotting: GFAP and MAP-2 (as above), DDHD2 (Proteintech, 25203-1-AP, RRID:AB_2879957), β-actin (Sigma-Aldrich, A5316; RRID:AB_476743), IRDye 680RD Goat anti-Mouse IgG Secondary Antibody (Licorbio, 926-68070, RRID:AB_10956588), IRDye 800CW Goat anti-Rabbit IgG Secondary Antibody (Licorbio, 926-32211, RRID:AB_621843), IRDye 800CW Donkey anti-Guinea Pig IgG Secondary Antibody (Licorbio, 926-32411, all from LicorBio, RRID:AB_1850024).

Validation

anti-GFAP validated by abcam for use in ICC/IF (mouse/rat), IHC (PFA fixed, mouse), IHC-P (mouse/rat), IP (mouse) and WB (Rat).; anti-TOMM20 validated by abcam for use in WB (Human), ICC/IF (Human), Flow Cyt (Intra, human), IHC-P (human/mouse/rat). Both GFAP and TOMM20 abcam antibodies have also been published in 1204 and 93 peer-reviewed articles respectively.; anti-MAP2 was validated by Synaptic systems for use in WB, ICC, IHC, IHC-P to recognize all four published isoforms.; anti-Synapsin1 antibody has been validated by Synaptic systems for use in WB, IP, ICC, IHC, IHC-P for specificity of Synapsin1a and 1b in human (P17600), rat (P09951), mouse (O88935). Also validated staining in Synapsin knockout cells by manufacturer. >38 peer-reviewed articles published use in WB and ICC and 1 article each for DNA-PAINT (rat), EM (rat) and FACS (mouse) and ELIZA (specices unspecified), although it remains untested by Synaptic systems for those purposes.; Invitrogen tested anti-Rabbit IgG-647 dilutions for IHC and ICC/IF, namely, immunofluorescence analysis was performed by manufacturer using HeLa cells stained with alpha Tubulin Rabbit Polyclonal Antibody and AlexaFlour phalloidin. It has also been independently validated in various published peer-reviewed articles. (>2000).; Anti-mouse-488 has been validated by Invitrogen for use in IHC, ICC/IF and Flow, namely, IF in Drosophila melanogaster embryos, Bovine pulmonary artery endothelial cells, HeLa cells, primary neurons from rat cerebellum, granule neurons and glial cells, and labeled paint probes hybridized to human metaphase chromosomes. It has also been independently validated in various published peer-reviewed articles. (>9000).; anti-Rabbit-Plus-647 IgG was validated by Invitrogen for use in WB, IHC-P, IHC-F, and ICC/IF, namely, IF in A549 cells against cells stained against ZO-1, PMP70, and PSD-95 in E18 Sparague Dawley primary cortical neuronal cells. It has also been independently validated in various published peer-reviewed articles. (>700).; anti-DDHD2 antibody was validated in our study against DDHD2 knockout background using western blot. Proteintech validated the antibody for use in WB (tested in mouse lung and testis and rat brain), IHC (mouse brain and cerebellum), and IFF/ICC (HEK-293). The antibody has also validated for above usage by various published applications, against human, mouse and rat DDHD2 protein; with additional applications for KO/KD and IP- that were not expressly tested by manufacturer.; anti-beta-actin was validated by Sigma Aldrich for use in IHC-F/IHC-P, and WB against cultured human and chicken fibroblast cell extracts and MDCK cell lysates.; IRdye antibodies 680RD (anti-Mouse), 800CW (anti-Rabbit) and 800CW (anti-Guinea Pig) were validated for Odyssey WB and In-cell western assay detections. They has also been independently validated in various published peer-reviewed articles, patents, PhD thesis and supplementary data. (>17000).

Eukaryotic cell lines

Policy information about cell lines and Sex and Gender in Research

Cell line source(s)

PC12 cells (ATCC, CRL-1721), HEK293T cells (ATCC, CRL-3216) and Gibco Viral Production Cells (Gibco, A35347) cell lines

Authentication

Describe the authentication procedures for each cell line used OR declare that none of the cell lines used were authenticated.

Mycoplasma contamination

PC12 cells (ATCC, CRL-1721), HEK293T cells (ATCC, CRL-3216) and Gibco Viral Production Cells (Gibco, A35347) cell lines were confirmed to be mycoplasma negative prior experiments.

Commonly misidentified lines
(See ICLAC register)

no misidentified cell lines were used.

Animals and other research organisms

Policy information about studies involving animals; ARRIVE guidelines recommended for reporting animal research, and Sex and Gender in Research

Laboratory animals

Mouse, C57BL6/J and DDHD2 knockout, E16 embryos (mixed female and male)

Wild animals	Not used
Reporting on sex	Findings do not apply to one sex. Female and male embryos were dissected and brain regions of interest mixed before extraction of neurons and glial cells. For the analysis of Acetyl CoA in brain lysates, samples were obtained from female mice.
Field-collected samples	n/a
Ethics oversight	All experiments performed under approval of University of Queensland animal ethics committee (AE000770, AE000209), and University of Helsinki under license number KEK21-012 and KEK24-013.

Note that full information on the approval of the study protocol must also be provided in the manuscript.

Plants

Seed stocks	n/a
Novel plant genotypes	n/a
Authentication	n/a
