# Supplementary material for: DDHD2 provides a flux of saturated fatty acids for neuronal energy and function
Source: Nat Metab. 2025 Sep 30;7(10):2117–41. doi: 10.1038/s42255-025-01367-x (PMC12552131; doi:10.1038/s42255-025-01367-x)
Supplement: Supplementary file 11 — Uncropped western blot images of Fig. 4i. [file 42255_2025_1367_MOESM11_ESM.pdf]

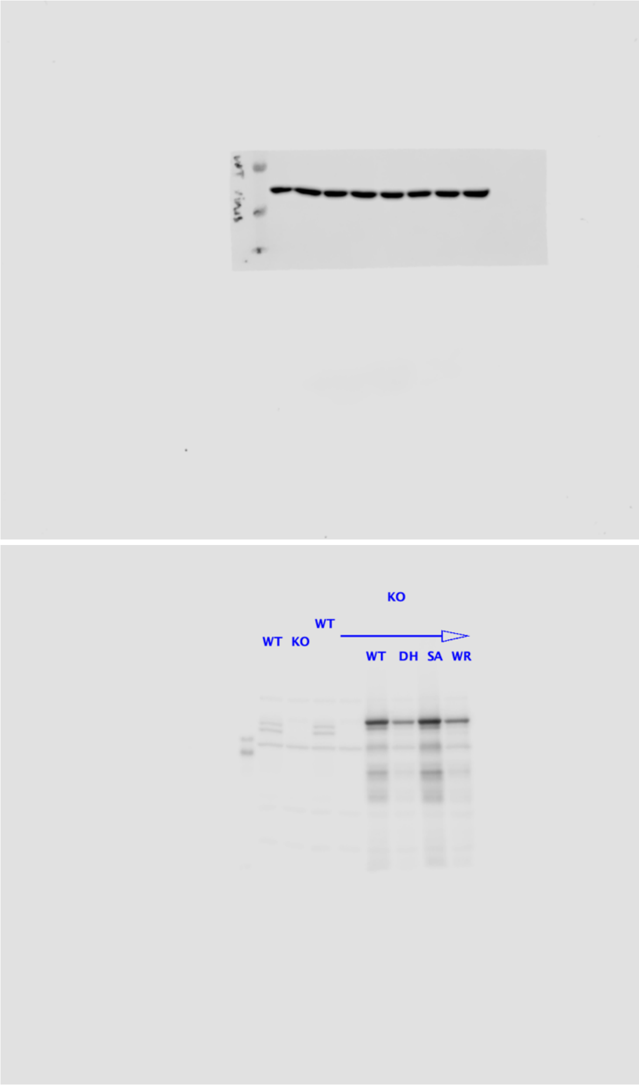

Uncropped Western Blot images of Fig. 4i. The blot above shows the actin staining and the blot below shows the DDHD2 staining, along with the markers on lane 1.
