# Supplementary figures and images for: DDHD2 provides a flux of saturated fatty acids for neuronal energy and function
Source: Nat Metab. 2025 Sep 30;7(10):2117–41. doi: 10.1038/s42255-025-01367-x (PMC12552131; doi:10.1038/s42255-025-01367-x)

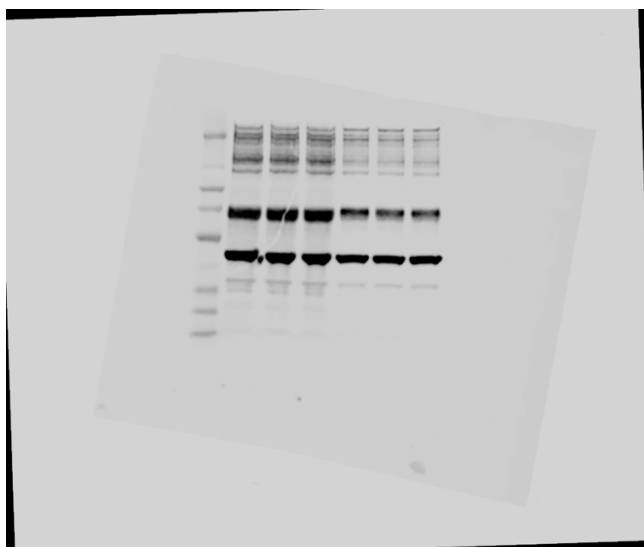

**Uncropped Western Blot image of Extended Data Fig. 1b.**

Supplement: Supplementary file 12 — Uncropped western blot image of Extended Data Fig. 1b. [file 42255_2025_1367_MOESM12_ESM.pdf]
